# Supplementary material for: DHSpred: support-vector-machine-based human DNase I hypersensitive sites prediction using the optimal features selected by random forest
Source: Oncotarget. 2017 Dec 8;9(2):1944–56. doi: 10.18632/oncotarget.23099 (PMC5788611; doi:10.18632/oncotarget.23099)
Supplement: Supplementary file 1 [file oncotarget-09-1944-s001.pdf]

# DHSpred: support-vector-machine-based human DNase I hypersensitive sites prediction using the optimal features selected by random forest

## SUPPLEMENTARY MATERIALS

### Guideline of the DHSpred webserver

For users' convenience, we have established a user-friendly webserver that implements the proposed DHSpred is shown in Supplementary Figure S1. The webserver is freely accessible to the public for academic use. This section aims to give researchers a step-by-step guideline on the usage of the webserver. The guideline is described as follows:

Step 1. Go to the website (http://thegleelab.org/DHSpred.html) to see the homepage of the webserver as shown in Supplementary Figure 1.

Step 2. Enter the query DNA sequences into the input box at the center of Supplementary Figure 1. You can also directly upload input file via the Browse button. The input sequences should be in the FASTA format. Examples of FASTA-formatted sequences can be seen by clicking on the Example button right above the input box.

Step 3. By clicking on the Submit button, you will get the predicted results on the screen of your computer. The first, the second, the third and the fourth column respectively represent serial number, user FASTA ID, DHS or non-DHS and probability of DHS.

Step 4. Click on the Reset button to clear the query sequences you enter to the input box.

Step 5. Click on the Dataset button to download the dataset used in this paper.

A

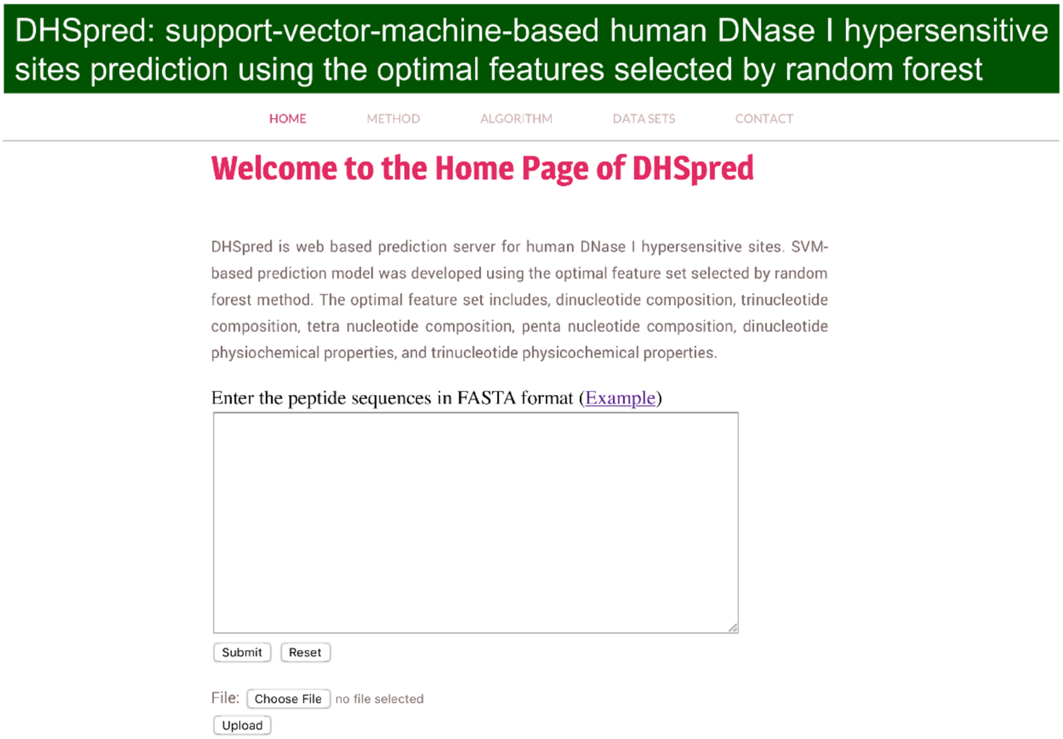


B

| S.NO | FASTA ID | DHS or Non-DHS | Prob |
|---|---|---|---|
| 1 | Pos1 | DHS | 0.7784 |
| 2 | Pos2 | DHS | 0.9660 |
| 3 | Neg1 | Non-DHS | 0.1014 |
| 4 | Neg2 | Non-DHS | 0.1223 |

**Supplementary Figure 1: Homepage of the online webserver that implements the proposed DHSpred.** (**A**) semi-screenshot for the top-page of the DHSpred web-server. (**B**) The prediction result of the four example sequences.

**Supplementary Table 1: Features used in this study along with their importance score.** See_ Supplementary_Table 1

**Supplementary Table 2: The values of six DNA dinucleotide physicochemical properties**

| | Twist | Tilt | Roll | Shift | Slide | Rise |
|---|---|---|---|---|---|---|
| AA | 35 | 0.10 | 1.40 | 0.06 | 0.16 | 3.28 |
| AC | 32 | 0.30 | 1.40 | 0.06 | 0.43 | 3.23 |
| AG | 28 | 0.20 | 5.50 | 0.06 | 0.34 | 3.27 |
| AT | 31 | 0.30 | 1.20 | 0.12 | 0.57 | 3.30 |
| CA | 43 | 0.00 | 1.20 | 0.02 | 1.88 | 3.32 |
| CC | 35 | 0.10 | 3.90 | 0.05 | 0.28 | 3.40 |
| CG | 31 | 0.00 | 6.20 | 0.06 | 0.68 | 3.25 |
| CT | 28 | 0.20 | 5.50 | 0.06 | 0.34 | 3.27 |
| GA | 41 | 0.00 | 0.40 | 0.00 | 0.01 | 3.43 |
| GC | 40 | 0.00 | 6.80 | 0.30 | 0.31 | 3.57 |
| GG | 35 | 0.10 | 3.90 | 0.05 | 0.28 | 3.40 |
| GT | 32 | 0.30 | 1.40 | 0.06 | 0.43 | 3.23 |
| TA | 43 | 1.40 | 0.60 | 0.17 | 0.38 | 3.37 |
| TC | 41 | 0.00 | 0.40 | 0.00 | 0.01 | 3.43 |
| TG | 43 | 0.00 | 1.20 | 0.02 | 1.88 | 3.32 |
| TT | 35 | 0.10 | 1.40 | 0.06 | 0.16 | 3.28 |

The first column represents the dinucleotides. The columns 2–6 represent the six physiochemical properties of the dinucleotides.

**Supplementary Table 3: The values of 12 DNA trinucleotide physicochemical properties.** See Supplementary_Table_3
