# Supplementary material for: DHSpred: support-vector-machine-based human DNase I hypersensitive sites prediction using the optimal features selected by random forest
Source: Oncotarget. 2017 Dec 8;9(2):1944–56. doi: 10.18632/oncotarget.23099 (PMC5788611; doi:10.18632/oncotarget.23099)
Supplement: Supplementary file 2 [file oncotarget-09-1944-s002.docx]

**Supplementary Table 1:** **Features used in this study along with their importance score.** The first, the second, the third, and the fourth column respectively contain serial number, composition, nucleotides belong to the composition, and feature importance score. The optimal feature set is highlighted in green.

| S. No | Composition | Nucleotides | FIS |
| --- | --- | --- | --- |
| 1 | MNC | A | 0.0003583 |
| 2 | MNC | C | 0.0003268 |
| 3 | MNC | T | 0.0003949 |
| 4 | MNC | G | 0.0003621 |
| 5 | DNC | AA | 0.0003738 |
| 6 | DNC | AC | 0.0003034 |
| 7 | DNC | GT | 0.0002978 |
| 8 | DNC | AG | 0.0002722 |
| 9 | DNC | CC | 0.0003489 |
| 10 | DNC | TT | 0.0004134 |
| 11 | DNC | CG | 0.0066341 |
| 12 | DNC | TC | 0.0002532 |
| 13 | DNC | GG | 0.0003271 |
| 14 | DNC | GC | 0.0016203 |
| 15 | DNC | AT | 0.0005594 |
| 16 | DNC | GA | 0.0002677 |
| 17 | DNC | TG | 0.0006785 |
| 18 | DNC | TA | 0.0007276 |
| 19 | DNC | CA | 0.0009681 |
| 20 | DNC | CT | 0.0002673 |
| 21 | TNC | ACC | 0.0002661 |
| 22 | TNC | ATG | 0.0004628 |
| 23 | TNC | AAG | 0.0002737 |
| 24 | TNC | AAA | 0.0003598 |
| 25 | TNC | ATC | 0.0002775 |
| 26 | TNC | AAC | 0.0003458 |
| 27 | TNC | ATA | 0.0002634 |
| 28 | TNC | AGG | 0.0002455 |
| 29 | TNC | CCT | 0.0002841 |
| 30 | TNC | CTC | 0.0003176 |
| 31 | TNC | AGC | 0.0003961 |
| 32 | TNC | ACA | 0.0005527 |
| 33 | TNC | AGA | 0.0002966 |
| 34 | TNC | CAT | 0.0004453 |
| 35 | TNC | AAT | 0.0003825 |
| 36 | TNC | ATT | 0.0005781 |
| 37 | TNC | CTG | 0.0006027 |
| 38 | TNC | CTA | 0.0004020 |
| 39 | TNC | ACT | 0.0003167 |
| 40 | TNC | CAC | 0.0005042 |
| 41 | TNC | ACG | 0.0007886 |
| 42 | TNC | CAA | 0.0003550 |
| 43 | TNC | AGT | 0.0002947 |
| 44 | TNC | CAG | 0.0006893 |
| 45 | TNC | CCG | 0.0027112 |
| 46 | TNC | CCC | 0.0002986 |
| 47 | TNC | TAT | 0.0003886 |
| 48 | TNC | GGT | 0.0002891 |
| 49 | TNC | TGT | 0.0004542 |
| 50 | TNC | CGA | 0.0013839 |
| 51 | TNC | CCA | 0.0003519 |
| 52 | TNC | TCT | 0.0002765 |
| 53 | TNC | GAT | 0.0003139 |
| 54 | TNC | CGG | 0.0023964 |
| 55 | TNC | CTT | 0.0003024 |
| 56 | TNC | TGC | 0.0003194 |
| 57 | TNC | GGG | 0.0002810 |
| 58 | TNC | TAG | 0.0004769 |
| 59 | TNC | GGA | 0.0002671 |
| 60 | TNC | TAA | 0.0003534 |
| 61 | TNC | GGC | 0.0004310 |
| 62 | TNC | TAC | 0.0002952 |
| 63 | TNC | TTC | 0.0002886 |
| 64 | TNC | TCG | 0.0011436 |
| 65 | TNC | TTT | 0.0002725 |
| 66 | TNC | TTG | 0.0003345 |
| 67 | TNC | TCC | 0.0002936 |
| 68 | TNC | GAA | 0.0003038 |
| 69 | TNC | TGG | 0.0003310 |
| 70 | TNC | GCA | 0.0004747 |
| 71 | TNC | GTA | 0.0002905 |
| 72 | TNC | GCC | 0.0002959 |
| 73 | TNC | GTC | 0.0002965 |
| 74 | TNC | TGA | 0.0003995 |
| 75 | TNC | GCG | 0.0056627 |
| 76 | TNC | GTG | 0.0003511 |
| 77 | TNC | GAG | 0.0003114 |
| 78 | TNC | GTT | 0.0003279 |
| 79 | TNC | GCT | 0.0002684 |
| 80 | TNC | TTA | 0.0005170 |
| 81 | TNC | GAC | 0.0002779 |
| 82 | TNC | CGT | 0.0007679 |
| 83 | TNC | TCA | 0.0003319 |
| 84 | TNC | CGC | 0.0034574 |
| 85 | TeNC | GTAC | 0.0002113 |
| 86 | TeNC | CGAG | 0.0007010 |
| 87 | TeNC | GTAA | 0.0002417 |
| 88 | TeNC | CGAA | 0.0002924 |
| 89 | TeNC | AAAT | 0.0003909 |
| 90 | TeNC | CGAC | 0.0006431 |
| 91 | TeNC | AGTG | 0.0003388 |
| 92 | TeNC | AGTA | 0.0002673 |
| 93 | TeNC | AGTC | 0.0004356 |
| 94 | TeNC | AAAA | 0.0003582 |
| 95 | TeNC | CGAT | 0.0002989 |
| 96 | TeNC | AAAC | 0.0003177 |
| 97 | TeNC | GAGC | 0.0003622 |
| 98 | TeNC | GTAT | 0.0001800 |
| 99 | TeNC | AGTT | 0.0004348 |
| 100 | TeNC | AACA | 0.0002420 |
| 101 | TeNC | GTAG | 0.0002952 |
| 102 | TeNC | GAAT | 0.0002923 |
| 103 | TeNC | TTAG | 0.0003612 |
| 104 | TeNC | TTAC | 0.0003246 |
| 105 | TeNC | TTAA | 0.0003726 |
| 106 | TeNC | GAAC | 0.0003217 |
| 107 | TeNC | GAAA | 0.0005047 |
| 108 | TeNC | GAAG | 0.0002783 |
| 109 | TeNC | TTAT | 0.0002930 |
| 110 | TeNC | CTAC | 0.0002748 |
| 111 | TeNC | GCTG | 0.0003190 |
| 112 | TeNC | GCTC | 0.0003756 |
| 113 | TeNC | GCTA | 0.0003187 |
| 114 | TeNC | ATAA | 0.0002619 |
| 115 | TeNC | ATAC | 0.0002470 |
| 116 | TeNC | CCAT | 0.0003037 |
| 117 | TeNC | ATAG | 0.0002477 |
| 118 | TeNC | GCTT | 0.0002581 |
| 119 | TeNC | CCAA | 0.0004641 |
| 120 | TeNC | ATTT | 0.0002700 |
| 121 | TeNC | CCAC | 0.0002971 |
| 122 | TeNC | ATAT | 0.0003983 |
| 123 | TeNC | CCAG | 0.0004333 |
| 124 | TeNC | CGCT | 0.0008798 |
| 125 | TeNC | TAAG | 0.0004838 |
| 126 | TeNC | TAAC | 0.0003102 |
| 127 | TeNC | TAAA | 0.0002514 |
| 128 | TeNC | TGTC | 0.0002739 |
| 129 | TeNC | TGTA | 0.0002665 |
| 130 | TeNC | TCTT | 0.0003242 |
| 131 | TeNC | TGTG | 0.0005713 |
| 132 | TeNC | TAAT | 0.0003362 |
| 133 | TeNC | TCTG | 0.0003906 |
| 134 | TeNC | ACTA | 0.0003106 |
| 135 | TeNC | ACTC | 0.0003090 |
| 136 | TeNC | TCTC | 0.0002805 |
| 137 | TeNC | TATG | 0.0002742 |
| 138 | TeNC | TCTA | 0.0002981 |
| 139 | TeNC | ACTG | 0.0004239 |
| 140 | TeNC | AAGG | 0.0003223 |
| 141 | TeNC | TAGT | 0.0002502 |
| 142 | TeNC | CGGA | 0.0007307 |
| 143 | TeNC | AAGC | 0.0003702 |
| 144 | TeNC | AAAG | 0.0002865 |
| 145 | TeNC | AAGA | 0.0002883 |
| 146 | TeNC | CTAA | 0.0004382 |
| 147 | TeNC | ACTT | 0.0002763 |
| 148 | TeNC | GTGC | 0.0003149 |
| 149 | TeNC | GTGG | 0.0002842 |
| 150 | TeNC | ACGA | 0.0003092 |
| 151 | TeNC | ACGC | 0.0005253 |
| 152 | TeNC | AAGT | 0.0003133 |
| 153 | TeNC | ACGG | 0.0002534 |
| 154 | TeNC | CGGT | 0.0004267 |
| 155 | TeNC | GGCC | 0.0003544 |
| 156 | TeNC | GGCA | 0.0003856 |
| 157 | TeNC | GTGT | 0.0003750 |
| 158 | TeNC | TTCT | 0.0002988 |
| 159 | TeNC | AGGG | 0.0002809 |
| 160 | TeNC | AGGA | 0.0002441 |
| 161 | TeNC | AGGC | 0.0002565 |
| 162 | TeNC | AATC | 0.0003484 |
| 163 | TeNC | GATT | 0.0003124 |
| 164 | TeNC | CGTG | 0.0002432 |
| 165 | TeNC | GTCG | 0.0004258 |
| 166 | TeNC | GACT | 0.0002787 |
| 167 | TeNC | ATCA | 0.0002705 |
| 168 | TeNC | AGGT | 0.0002556 |
| 169 | TeNC | TTCG | 0.0003036 |
| 170 | TeNC | AATA | 0.0002749 |
| 171 | TeNC | TTCA | 0.0004141 |
| 172 | TeNC | TTCC | 0.0003186 |
| 173 | TeNC | GACA | 0.0003036 |
| 174 | TeNC | GACC | 0.0003680 |
| 175 | TeNC | GATG | 0.0005795 |
| 176 | TeNC | GATA | 0.0003077 |
| 177 | TeNC | GACG | 0.0009301 |
| 178 | TeNC | GATC | 0.0002791 |
| 179 | TeNC | GCCG | 0.0013720 |
| 180 | TeNC | AGAC | 0.0002404 |
| 181 | TeNC | ATCT | 0.0002819 |
| 182 | TeNC | GCCC | 0.0002627 |
| 183 | TeNC | GCCA | 0.0003006 |
| 184 | TeNC | CCGG | 0.0015380 |
| 185 | TeNC | TACT | 0.0002396 |
| 186 | TeNC | TCAG | 0.0002559 |
| 187 | TeNC | CCGC | 0.0018306 |
| 188 | TeNC | CAGT | 0.0002803 |
| 189 | TeNC | CCTT | 0.0003630 |
| 190 | TeNC | CACG | 0.0002762 |
| 191 | TeNC | ATCG | 0.0002952 |
| 192 | TeNC | TAGG | 0.0003883 |
| 193 | TeNC | ATCC | 0.0003561 |
| 194 | TeNC | CTCA | 0.0003544 |
| 195 | TeNC | CAGA | 0.0003521 |
| 196 | TeNC | CCTC | 0.0002481 |
| 197 | TeNC | CAGC | 0.0004381 |
| 198 | TeNC | CCGT | 0.0003322 |
| 199 | TeNC | GGCT | 0.0002743 |
| 200 | TeNC | CCTG | 0.0005665 |
| 201 | TeNC | CAGG | 0.0004166 |
| 202 | TeNC | TGGG | 0.0003024 |
| 203 | TeNC | CTCC | 0.0002709 |
| 204 | TeNC | TGGC | 0.0003351 |
| 205 | TeNC | TGGA | 0.0004189 |
| 206 | TeNC | TCGC | 0.0010689 |
| 207 | TeNC | TCGA | 0.0003285 |
| 208 | TeNC | TCGG | 0.0003797 |
| 209 | TeNC | TGGT | 0.0002551 |
| 210 | TeNC | GCCT | 0.0002777 |
| 211 | TeNC | CACA | 0.0006058 |
| 212 | TeNC | TGTT | 0.0003218 |
| 213 | TeNC | TCGT | 0.0002018 |
| 214 | TeNC | TAGA | 0.0002665 |
| 215 | TeNC | ACAG | 0.0003350 |
| 216 | TeNC | CGGC | 0.0009248 |
| 217 | TeNC | ACAC | 0.0008694 |
| 218 | TeNC | ATGT | 0.0003174 |
| 219 | TeNC | ACAA | 0.0002599 |
| 220 | TeNC | CTAG | 0.0003877 |
| 221 | TeNC | TAGC | 0.0003593 |
| 222 | TeNC | ACAT | 0.0002752 |
| 223 | TeNC | CGGG | 0.0005429 |
| 224 | TeNC | ACGT | 0.0005454 |
| 225 | TeNC | GGAA | 0.0003729 |
| 226 | TeNC | GGAC | 0.0002868 |
| 227 | TeNC | CCTA | 0.0003250 |
| 228 | TeNC | AGAG | 0.0002865 |
| 229 | TeNC | GGAG | 0.0003060 |
| 230 | TeNC | ATTG | 0.0005741 |
| 231 | TeNC | CTCT | 0.0002436 |
| 232 | TeNC | ATTC | 0.0003617 |
| 233 | TeNC | GGAT | 0.0003777 |
| 234 | TeNC | ATTA | 0.0002997 |
| 235 | TeNC | GTGA | 0.0003110 |
| 236 | TeNC | TACG | 0.0003942 |
| 237 | TeNC | CTTG | 0.0004028 |
| 238 | TeNC | TGCG | 0.0013054 |
| 239 | TeNC | TGAT | 0.0003400 |
| 240 | TeNC | TTTA | 0.0003450 |
| 241 | TeNC | TTTC | 0.0002537 |
| 242 | TeNC | TTTG | 0.0002671 |
| 243 | TeNC | CAAG | 0.0002773 |
| 244 | TeNC | AACG | 0.0005156 |
| 245 | TeNC | GCAG | 0.0006692 |
| 246 | TeNC | AGAT | 0.0004800 |
| 247 | TeNC | GCAA | 0.0004000 |
| 248 | TeNC | CAAA | 0.0002961 |
| 249 | TeNC | TGAG | 0.0003351 |
| 250 | TeNC | TTTT | 0.0002952 |
| 251 | TeNC | TGAA | 0.0002785 |
| 252 | TeNC | TGAC | 0.0002942 |
| 253 | TeNC | GCAT | 0.0004740 |
| 254 | TeNC | CAAT | 0.0005357 |
| 255 | TeNC | CTGT | 0.0003143 |
| 256 | TeNC | GTCT | 0.0003078 |
| 257 | TeNC | TCAT | 0.0003993 |
| 258 | TeNC | AATT | 0.0004532 |
| 259 | TeNC | CGTT | 0.0004469 |
| 260 | TeNC | CTCG | 0.0006756 |
| 261 | TeNC | TATA | 0.0002514 |
| 262 | TeNC | CTAT | 0.0004241 |
| 263 | TeNC | TATC | 0.0008042 |
| 264 | TeNC | TCAA | 0.0002891 |
| 265 | TeNC | TCAC | 0.0002818 |
| 266 | TeNC | AATG | 0.0002775 |
| 267 | TeNC | CTGC | 0.0003501 |
| 268 | TeNC | TATT | 0.0003121 |
| 269 | TeNC | CGTC | 0.0012696 |
| 270 | TeNC | CGTA | 0.0002854 |
| 271 | TeNC | GTTA | 0.0002736 |
| 272 | TeNC | GTTC | 0.0004675 |
| 273 | TeNC | GGGT | 0.0002461 |
| 274 | TeNC | CCCC | 0.0003096 |
| 275 | TeNC | GTCA | 0.0003291 |
| 276 | TeNC | GTTG | 0.0003596 |
| 277 | TeNC | GTCC | 0.0003840 |
| 278 | TeNC | AACC | 0.0003644 |
| 279 | TeNC | ACCG | 0.0005994 |
| 280 | TeNC | ACCA | 0.0002605 |
| 281 | TeNC | ACCC | 0.0003157 |
| 282 | TeNC | GGGG | 0.0004060 |
| 283 | TeNC | GGGC | 0.0003940 |
| 284 | TeNC | GTTT | 0.0002983 |
| 285 | TeNC | GGGA | 0.0003257 |
| 286 | TeNC | ACCT | 0.0003719 |
| 287 | TeNC | CTTT | 0.0002735 |
| 288 | TeNC | AACT | 0.0003182 |
| 289 | TeNC | GAGT | 0.0003868 |
| 290 | TeNC | GGTA | 0.0002865 |
| 291 | TeNC | GGTG | 0.0002619 |
| 292 | TeNC | AGCA | 0.0003671 |
| 293 | TeNC | GGCG | 0.0017976 |
| 294 | TeNC | AGCC | 0.0002927 |
| 295 | TeNC | TTGT | 0.0003465 |
| 296 | TeNC | AGCG | 0.0010640 |
| 297 | TeNC | GAGG | 0.0002770 |
| 298 | TeNC | GAGA | 0.0003472 |
| 299 | TeNC | GGTT | 0.0002692 |
| 300 | TeNC | TTGA | 0.0003145 |
| 301 | TeNC | TTGC | 0.0004059 |
| 302 | TeNC | CTGG | 0.0004729 |
| 303 | TeNC | AGCT | 0.0003964 |
| 304 | TeNC | TTGG | 0.0003023 |
| 305 | TeNC | GGTC | 0.0002799 |
| 306 | TeNC | CACT | 0.0002722 |
| 307 | TeNC | ATGA | 0.0003571 |
| 308 | TeNC | ATGG | 0.0002481 |
| 309 | TeNC | TGCT | 0.0003542 |
| 310 | TeNC | CCCT | 0.0003567 |
| 311 | TeNC | GCGC | 0.0021925 |
| 312 | TeNC | CAAC | 0.0005910 |
| 313 | TeNC | GCGA | 0.0013397 |
| 314 | TeNC | GCGG | 0.0016822 |
| 315 | TeNC | ATGC | 0.0003951 |
| 316 | TeNC | TGCC | 0.0003374 |
| 317 | TeNC | CCCA | 0.0002867 |
| 318 | TeNC | TGCA | 0.0004769 |
| 319 | TeNC | CCCG | 0.0006591 |
| 320 | TeNC | GCAC | 0.0003593 |
| 321 | TeNC | CACC | 0.0004883 |
| 322 | TeNC | TACA | 0.0002776 |
| 323 | TeNC | TACC | 0.0002881 |
| 324 | TeNC | CTTA | 0.0004494 |
| 325 | TeNC | GCGT | 0.0008325 |
| 326 | TeNC | CATA | 0.0003300 |
| 327 | TeNC | CATC | 0.0004173 |
| 328 | TeNC | CCGA | 0.0003060 |
| 329 | TeNC | CATG | 0.0002703 |
| 330 | TeNC | TCCT | 0.0002952 |
| 331 | TeNC | AGAA | 0.0003793 |
| 332 | TeNC | CATT | 0.0002901 |
| 333 | TeNC | CTTC | 0.0003544 |
| 334 | TeNC | CTGA | 0.0003243 |
| 335 | TeNC | CGCG | 0.0027417 |
| 336 | TeNC | TCCG | 0.0006690 |
| 337 | TeNC | CGCC | 0.0023041 |
| 338 | TeNC | TCCC | 0.0003043 |
| 339 | TeNC | CGCA | 0.0008314 |
| 340 | TeNC | TCCA | 0.0003147 |
| 341 | PNC | GCGTT | 0.0004607 |
| 342 | PNC | AAATG | 0.0004128 |
| 343 | PNC | GCCCG | 0.0003315 |
| 344 | PNC | GCCCA | 0.0002202 |
| 345 | PNC | AAATC | 0.0002880 |
| 346 | PNC | GCCCC | 0.0003263 |
| 347 | PNC | AAATA | 0.0002661 |
| 348 | PNC | CCTAT | 0.0002028 |
| 349 | PNC | TTTCT | 0.0002592 |
| 350 | PNC | GCCCT | 0.0002425 |
| 351 | PNC | AAATT | 0.0006422 |
| 352 | PNC | GCGTG | 0.0002516 |
| 353 | PNC | GCGTA | 0.0002690 |
| 354 | PNC | GCGTC | 0.0005684 |
| 355 | PNC | CCTAA | 0.0003559 |
| 356 | PNC | CCTAC | 0.0001929 |
| 357 | PNC | CCTAG | 0.0002653 |
| 358 | PNC | TGGTG | 0.0002030 |
| 359 | PNC | AGTCT | 0.0003632 |
| 360 | PNC | GTGGG | 0.0002724 |
| 361 | PNC | TGGTC | 0.0002015 |
| 362 | PNC | GTGGA | 0.0003128 |
| 363 | PNC | TGGTA | 0.0001297 |
| 364 | PNC | GTGGC | 0.0002781 |
| 365 | PNC | AGACA | 0.0002274 |
| 366 | PNC | TTACT | 0.0001782 |
| 367 | PNC | AGACC | 0.0003901 |
| 368 | PNC | GTAGA | 0.0001917 |
| 369 | PNC | CGCAG | 0.0004374 |
| 370 | PNC | GATAC | 0.0002684 |
| 371 | PNC | AGACG | 0.0002172 |
| 372 | PNC | GATAA | 0.0004083 |
| 373 | PNC | GTGGT | 0.0001881 |
| 374 | PNC | TGGTT | 0.0002745 |
| 375 | PNC | CCCCA | 0.0004197 |
| 376 | PNC | TTACG | 0.0001831 |
| 377 | PNC | TTACA | 0.0002059 |
| 378 | PNC | AGACT | 0.0002443 |
| 379 | PNC | TTACC | 0.0002113 |
| 380 | PNC | CGCAT | 0.0001542 |
| 381 | PNC | AGCAA | 0.0002518 |
| 382 | PNC | TTCAT | 0.0003542 |
| 383 | PNC | AGCAC | 0.0003326 |
| 384 | PNC | TCGCA | 0.0002104 |
| 385 | PNC | GAGAC | 0.0002966 |
| 386 | PNC | TCGCG | 0.0006765 |
| 387 | PNC | AGCAG | 0.0008768 |
| 388 | PNC | GTTTA | 0.0002999 |
| 389 | PNC | CCAAA | 0.0006249 |
| 390 | PNC | TTCAG | 0.0003494 |
| 391 | PNC | TTCAA | 0.0003451 |
| 392 | PNC | AGCAT | 0.0002898 |
| 393 | PNC | TTCAC | 0.0002424 |
| 394 | PNC | GCACT | 0.0002607 |
| 395 | PNC | TGTTT | 0.0002270 |
| 396 | PNC | TCATG | 0.0003979 |
| 397 | PNC | GAAAC | 0.0002686 |
| 398 | PNC | GAAAA | 0.0003743 |
| 399 | PNC | GTCGA | 0.0001842 |
| 400 | PNC | AACAG | 0.0002242 |
| 401 | PNC | TATAT | 0.0004991 |
| 402 | PNC | GACTT | 0.0001968 |
| 403 | PNC | GGGAT | 0.0002085 |
| 404 | PNC | AACAC | 0.0003423 |
| 405 | PNC | AACAA | 0.0001837 |
| 406 | PNC | TCATT | 0.0002670 |
| 407 | PNC | TGTTG | 0.0008186 |
| 408 | PNC | TGTTA | 0.0001347 |
| 409 | PNC | TGTTC | 0.0003125 |
| 410 | PNC | GACTG | 0.0002509 |
| 411 | PNC | TATAG | 0.0001448 |
| 412 | PNC | AACAT | 0.0001440 |
| 413 | PNC | TATAA | 0.0001419 |
| 414 | PNC | GACTC | 0.0002132 |
| 415 | PNC | TATAC | 0.0000654 |
| 416 | PNC | GACTA | 0.0001881 |
| 417 | PNC | CTAGC | 0.0002654 |
| 418 | PNC | CTAGA | 0.0002453 |
| 419 | PNC | CTAGG | 0.0003767 |
| 420 | PNC | CCTTT | 0.0002584 |
| 421 | PNC | GCACC | 0.0002551 |
| 422 | PNC | ACTCG | 0.0001672 |
| 423 | PNC | CCTTC | 0.0004130 |
| 424 | PNC | CCTTA | 0.0003431 |
| 425 | PNC | GAATC | 0.0001790 |
| 426 | PNC | CCTTG | 0.0004797 |
| 427 | PNC | CTAGT | 0.0002051 |
| 428 | PNC | TAATG | 0.0001783 |
| 429 | PNC | TGGCG | 0.0002359 |
| 430 | PNC | TAATC | 0.0002836 |
| 431 | PNC | TGGCC | 0.0002553 |
| 432 | PNC | TAATA | 0.0002610 |
| 433 | PNC | TGGCA | 0.0003100 |
| 434 | PNC | TGGCT | 0.0003893 |
| 435 | PNC | TAATT | 0.0002172 |
| 436 | PNC | GCTTG | 0.0002549 |
| 437 | PNC | AATGT | 0.0002629 |
| 438 | PNC | GCTTC | 0.0001849 |
| 439 | PNC | GCTTA | 0.0003421 |
| 440 | PNC | CGGCA | 0.0006370 |
| 441 | PNC | CACTC | 0.0002252 |
| 442 | PNC | CGGCC | 0.0002487 |
| 443 | PNC | CACTA | 0.0002218 |
| 444 | PNC | CACTG | 0.0004083 |
| 445 | PNC | CGGCG | 0.0006240 |
| 446 | PNC | AATGG | 0.0001876 |
| 447 | PNC | GGCTG | 0.0003079 |
| 448 | PNC | AATGC | 0.0003868 |
| 449 | PNC | AATGA | 0.0002452 |
| 450 | PNC | CGGCT | 0.0007271 |
| 451 | PNC | CACTT | 0.0002957 |
| 452 | PNC | GGCTA | 0.0001534 |
| 453 | PNC | GGGCT | 0.0002932 |
| 454 | PNC | ACTAT | 0.0001427 |
| 455 | PNC | TGACT | 0.0002875 |
| 456 | PNC | ATACA | 0.0003404 |
| 457 | PNC | ATACC | 0.0001666 |
| 458 | PNC | ATACG | 0.0000776 |
| 459 | PNC | ACTAC | 0.0003773 |
| 460 | PNC | GGGCG | 0.0017522 |
| 461 | PNC | ACTAA | 0.0004356 |
| 462 | PNC | GGGCA | 0.0004269 |
| 463 | PNC | ACTAG | 0.0001731 |
| 464 | PNC | GGGCC | 0.0003467 |
| 465 | PNC | GATAG | 0.0002755 |
| 466 | PNC | TGACG | 0.0003606 |
| 467 | PNC | ATACT | 0.0001928 |
| 468 | PNC | TGACA | 0.0002436 |
| 469 | PNC | TGACC | 0.0003276 |
| 470 | PNC | AGTTT | 0.0003112 |
| 471 | PNC | GCCGA | 0.0001768 |
| 472 | PNC | GCCGC | 0.0008163 |
| 473 | PNC | AGGTT | 0.0001945 |
| 474 | PNC | CCCCT | 0.0002344 |
| 475 | PNC | AGTTA | 0.0002976 |
| 476 | PNC | AGTTC | 0.0004174 |
| 477 | PNC | TGAGC | 0.0003115 |
| 478 | PNC | AGTTG | 0.0002329 |
| 479 | PNC | AGGTC | 0.0002143 |
| 480 | PNC | AGGTA | 0.0001389 |
| 481 | PNC | AACGG | 0.0002162 |
| 482 | PNC | TAACG | 0.0004148 |
| 483 | PNC | TAACC | 0.0003687 |
| 484 | PNC | TAACA | 0.0001293 |
| 485 | PNC | GTGCA | 0.0002889 |
| 486 | PNC | ACGCG | 0.0006188 |
| 487 | PNC | GTGCC | 0.0003301 |
| 488 | PNC | CGATT | 0.0001476 |
| 489 | PNC | GTGCG | 0.0002871 |
| 490 | PNC | TAACT | 0.0001930 |
| 491 | PNC | CACAA | 0.0003391 |
| 492 | PNC | AACGC | 0.0005007 |
| 493 | PNC | CACAC | 0.0005525 |
| 494 | PNC | CGATC | 0.0002844 |
| 495 | PNC | CGATA | 0.0000880 |
| 496 | PNC | GTGCT | 0.0003009 |
| 497 | PNC | GTGTG | 0.0003656 |
| 498 | PNC | GCTCG | 0.0002526 |
| 499 | PNC | ACATC | 0.0001961 |
| 500 | PNC | ATGAT | 0.0002590 |
| 501 | PNC | GCTCC | 0.0002849 |
| 502 | PNC | ACATG | 0.0002008 |
| 503 | PNC | CTGCT | 0.0003016 |
| 504 | PNC | TCTTA | 0.0001892 |
| 505 | PNC | TCTTC | 0.0002774 |
| 506 | PNC | ACCCT | 0.0002432 |
| 507 | PNC | ATGAA | 0.0002325 |
| 508 | PNC | GCTCT | 0.0003243 |
| 509 | PNC | ATGAC | 0.0001984 |
| 510 | PNC | ACATT | 0.0002775 |
| 511 | PNC | GGCGC | 0.0015663 |
| 512 | PNC | ATGAG | 0.0001976 |
| 513 | PNC | ACCCC | 0.0004361 |
| 514 | PNC | AATTG | 0.0004150 |
| 515 | PNC | ACCCA | 0.0002048 |
| 516 | PNC | CTGCG | 0.0008566 |
| 517 | PNC | TCTTT | 0.0002370 |
| 518 | PNC | GATAT | 0.0001540 |
| 519 | PNC | CTGCC | 0.0004100 |
| 520 | PNC | CGTAT | 0.0001553 |
| 521 | PNC | GGTGG | 0.0002510 |
| 522 | PNC | TTATC | 0.0004691 |
| 523 | PNC | GGTGC | 0.0002890 |
| 524 | PNC | TCGCC | 0.0001840 |
| 525 | PNC | GGTGA | 0.0001846 |
| 526 | PNC | TAGCT | 0.0001755 |
| 527 | PNC | ACTTT | 0.0001910 |
| 528 | PNC | ATGCT | 0.0004316 |
| 529 | PNC | GGTGT | 0.0001657 |
| 530 | PNC | CGTAG | 0.0004493 |
| 531 | PNC | AGGTG | 0.0002168 |
| 532 | PNC | CGTAA | 0.0001519 |
| 533 | PNC | CGTAC | 0.0000877 |
| 534 | PNC | TGAGT | 0.0004733 |
| 535 | PNC | ACTTG | 0.0002542 |
| 536 | PNC | TAGCG | 0.0002308 |
| 537 | PNC | ACTTA | 0.0001868 |
| 538 | PNC | TAGCA | 0.0003075 |
| 539 | PNC | ACTTC | 0.0002810 |
| 540 | PNC | TAGCC | 0.0005297 |
| 541 | PNC | TGAGA | 0.0002832 |
| 542 | PNC | ATCTT | 0.0002175 |
| 543 | PNC | GTAAT | 0.0002337 |
| 544 | PNC | AGAAT | 0.0002760 |
| 545 | PNC | TGAGG | 0.0002392 |
| 546 | PNC | CAACT | 0.0002817 |
| 547 | PNC | ATCTG | 0.0004248 |
| 548 | PNC | GTAAA | 0.0001694 |
| 549 | PNC | GTAAC | 0.0001632 |
| 550 | PNC | ATCTC | 0.0002213 |
| 551 | PNC | ATCTA | 0.0001939 |
| 552 | PNC | GTAAG | 0.0003805 |
| 553 | PNC | CAACG | 0.0002709 |
| 554 | PNC | CAACA | 0.0003232 |
| 555 | PNC | CAACC | 0.0003783 |
| 556 | PNC | CCAAT | 0.0007961 |
| 557 | PNC | GGAGC | 0.0003105 |
| 558 | PNC | AGGAG | 0.0002364 |
| 559 | PNC | TATGT | 0.0002647 |
| 560 | PNC | AGGAA | 0.0003272 |
| 561 | PNC | AGGAC | 0.0002196 |
| 562 | PNC | ATGTT | 0.0003744 |
| 563 | PNC | TCTAC | 0.0001376 |
| 564 | PNC | TGGGC | 0.0003017 |
| 565 | PNC | TCTAA | 0.0002025 |
| 566 | PNC | TGGGA | 0.0002917 |
| 567 | PNC | TCTAG | 0.0005951 |
| 568 | PNC | TGGGG | 0.0003087 |
| 569 | PNC | ATGTC | 0.0001828 |
| 570 | PNC | AGGAT | 0.0003215 |
| 571 | PNC | ATGTA | 0.0002723 |
| 572 | PNC | CTCAA | 0.0002642 |
| 573 | PNC | ATGTG | 0.0005212 |
| 574 | PNC | GGAGT | 0.0002156 |
| 575 | PNC | TGGGT | 0.0002046 |
| 576 | PNC | TCTAT | 0.0002963 |
| 577 | PNC | ACAAC | 0.0001552 |
| 578 | PNC | AAGTA | 0.0002232 |
| 579 | PNC | TTAGC | 0.0006422 |
| 580 | PNC | AAGTC | 0.0003236 |
| 581 | PNC | ACAAG | 0.0001983 |
| 582 | PNC | TTAGG | 0.0002371 |
| 583 | PNC | AAGTG | 0.0003923 |
| 584 | PNC | GTTAA | 0.0001743 |
| 585 | PNC | GTTAC | 0.0001865 |
| 586 | PNC | ATCCA | 0.0002821 |
| 587 | PNC | GGAGA | 0.0003852 |
| 588 | PNC | AATCT | 0.0001905 |
| 589 | PNC | GAGCC | 0.0003369 |
| 590 | PNC | ACGAA | 0.0001778 |
| 591 | PNC | ATTCG | 0.0000984 |
| 592 | PNC | ATTCA | 0.0006555 |
| 593 | PNC | TTAGT | 0.0001772 |
| 594 | PNC | ATTCC | 0.0003215 |
| 595 | PNC | ACAAT | 0.0001791 |
| 596 | PNC | AATCC | 0.0004110 |
| 597 | PNC | AATCA | 0.0003330 |
| 598 | PNC | AATCG | 0.0002061 |
| 599 | PNC | GTCGT | 0.0002761 |
| 600 | PNC | TGCGT | 0.0002578 |
| 601 | PNC | TTGGC | 0.0003965 |
| 602 | PNC | TTGGA | 0.0004516 |
| 603 | PNC | TTGGG | 0.0002309 |
| 604 | PNC | GTCTT | 0.0004336 |
| 605 | PNC | TGCGG | 0.0006377 |
| 606 | PNC | ACGAG | 0.0004007 |
| 607 | PNC | TGCGA | 0.0001629 |
| 608 | PNC | GTCTA | 0.0002100 |
| 609 | PNC | GGGGC | 0.0007074 |
| 610 | PNC | GTCTG | 0.0003441 |
| 611 | PNC | TTGGT | 0.0002146 |
| 612 | PNC | TAGGA | 0.0002677 |
| 613 | PNC | TAGGC | 0.0002893 |
| 614 | PNC | CCAAG | 0.0002070 |
| 615 | PNC | TAGGG | 0.0002975 |
| 616 | PNC | AGATT | 0.0003671 |
| 617 | PNC | CCGCT | 0.0002692 |
| 618 | PNC | TGCAC | 0.0003729 |
| 619 | PNC | TAGGT | 0.0001552 |
| 620 | PNC | TCCAG | 0.0002936 |
| 621 | PNC | ACGCC | 0.0007778 |
| 622 | PNC | CCGCG | 0.0008129 |
| 623 | PNC | AGATG | 0.0003246 |
| 624 | PNC | CCGCA | 0.0004828 |
| 625 | PNC | AGATA | 0.0003002 |
| 626 | PNC | CCGCC | 0.0011740 |
| 627 | PNC | AGATC | 0.0003324 |
| 628 | PNC | CTATA | 0.0002547 |
| 629 | PNC | CGGTT | 0.0001353 |
| 630 | PNC | TAAGC | 0.0002506 |
| 631 | PNC | TAAGA | 0.0007695 |
| 632 | PNC | TAAGG | 0.0002561 |
| 633 | PNC | CGGTG | 0.0002648 |
| 634 | PNC | CGGTA | 0.0001701 |
| 635 | PNC | TAAGT | 0.0002540 |
| 636 | PNC | CGGTC | 0.0002665 |
| 637 | PNC | AAGAC | 0.0001893 |
| 638 | PNC | GAAAG | 0.0003590 |
| 639 | PNC | AAGAA | 0.0004107 |
| 640 | PNC | GTCCG | 0.0006213 |
| 641 | PNC | AAGAG | 0.0002900 |
| 642 | PNC | CGAAC | 0.0001270 |
| 643 | PNC | ATAAC | 0.0001526 |
| 644 | PNC | GCTGC | 0.0003145 |
| 645 | PNC | GCTGA | 0.0003819 |
| 646 | PNC | GCTGG | 0.0002760 |
| 647 | PNC | CCCTC | 0.0002384 |
| 648 | PNC | CGAAT | 0.0001516 |
| 649 | PNC | ATAAA | 0.0002478 |
| 650 | PNC | AAGAT | 0.0002357 |
| 651 | PNC | CCCTT | 0.0002054 |
| 652 | PNC | TCATA | 0.0002782 |
| 653 | PNC | GCTGT | 0.0003124 |
| 654 | PNC | CTGGA | 0.0002775 |
| 655 | PNC | CTGGC | 0.0005643 |
| 656 | PNC | TCATC | 0.0002564 |
| 657 | PNC | CTGGG | 0.0002883 |
| 658 | PNC | ACCGG | 0.0003003 |
| 659 | PNC | GGTCC | 0.0002746 |
| 660 | PNC | GGTCA | 0.0002596 |
| 661 | PNC | ACCGC | 0.0004079 |
| 662 | PNC | GGTCG | 0.0002669 |
| 663 | PNC | ACCGA | 0.0002670 |
| 664 | PNC | CTATT | 0.0002667 |
| 665 | PNC | CTGGT | 0.0003484 |
| 666 | PNC | ACCGT | 0.0002198 |
| 667 | PNC | GGTCT | 0.0002427 |
| 668 | PNC | GCATG | 0.0002453 |
| 669 | PNC | TTAGA | 0.0001492 |
| 670 | PNC | TACAT | 0.0001793 |
| 671 | PNC | ATTCT | 0.0002239 |
| 672 | PNC | TGTAG | 0.0002222 |
| 673 | PNC | CCTGG | 0.0007465 |
| 674 | PNC | TGTAC | 0.0001736 |
| 675 | PNC | ACAAA | 0.0001932 |
| 676 | PNC | TGTAA | 0.0001906 |
| 677 | PNC | CCTGA | 0.0002561 |
| 678 | PNC | TACAC | 0.0003374 |
| 679 | PNC | CTCAC | 0.0004116 |
| 680 | PNC | TACAA | 0.0004065 |
| 681 | PNC | TACAG | 0.0002816 |
| 682 | PNC | CCTGT | 0.0003157 |
| 683 | PNC | TGTAT | 0.0001461 |
| 684 | PNC | CAAGA | 0.0002593 |
| 685 | PNC | GAGCT | 0.0006906 |
| 686 | PNC | CAAGC | 0.0002909 |
| 687 | PNC | GGACT | 0.0001734 |
| 688 | PNC | AGTCA | 0.0002361 |
| 689 | PNC | CAAGG | 0.0002953 |
| 690 | PNC | AGTCC | 0.0002515 |
| 691 | PNC | GCGAG | 0.0009730 |
| 692 | PNC | TACTA | 0.0002569 |
| 693 | PNC | CATAA | 0.0001641 |
| 694 | PNC | GCGAC | 0.0002674 |
| 695 | PNC | CATAG | 0.0002159 |
| 696 | PNC | GCGAA | 0.0003361 |
| 697 | PNC | GGACG | 0.0003416 |
| 698 | PNC | GAAAT | 0.0004721 |
| 699 | PNC | GGACC | 0.0004548 |
| 700 | PNC | CAAGT | 0.0002476 |
| 701 | PNC | GGACA | 0.0002291 |
| 702 | PNC | CGCAC | 0.0003168 |
| 703 | PNC | ACGCT | 0.0001498 |
| 704 | PNC | GCGAT | 0.0001374 |
| 705 | PNC | CATAT | 0.0002537 |
| 706 | PNC | ACTGA | 0.0002502 |
| 707 | PNC | GAAGT | 0.0003036 |
| 708 | PNC | AACGT | 0.0003200 |
| 709 | PNC | ACTGC | 0.0003087 |
| 710 | PNC | ATTGT | 0.0001706 |
| 711 | PNC | CGAAA | 0.0002993 |
| 712 | PNC | GAAGG | 0.0002642 |
| 713 | PNC | GAAGA | 0.0002131 |
| 714 | PNC | GTTAG | 0.0001417 |
| 715 | PNC | GAAGC | 0.0002108 |
| 716 | PNC | ATTGA | 0.0001757 |
| 717 | PNC | AGAGA | 0.0002513 |
| 718 | PNC | ATTGC | 0.0005509 |
| 719 | PNC | GCTAC | 0.0002670 |
| 720 | PNC | AACGA | 0.0001327 |
| 721 | PNC | ATTGG | 0.0012013 |
| 722 | PNC | GAGGG | 0.0002514 |
| 723 | PNC | ATCCT | 0.0003655 |
| 724 | PNC | GAGGC | 0.0002419 |
| 725 | PNC | GAGGA | 0.0002393 |
| 726 | PNC | GTATT | 0.0001437 |
| 727 | PNC | TTGCT | 0.0002092 |
| 728 | PNC | ACGAT | 0.0002423 |
| 729 | PNC | TGCCT | 0.0004028 |
| 730 | PNC | GTATC | 0.0001302 |
| 731 | PNC | ATCCG | 0.0002540 |
| 732 | PNC | GTATA | 0.0000697 |
| 733 | PNC | GAGGT | 0.0002048 |
| 734 | PNC | GTATG | 0.0001707 |
| 735 | PNC | TCCAA | 0.0002288 |
| 736 | PNC | TCCAC | 0.0002618 |
| 737 | PNC | TTGCG | 0.0003590 |
| 738 | PNC | AAGTT | 0.0007392 |
| 739 | PNC | ACGAC | 0.0002409 |
| 740 | PNC | TGCCA | 0.0002245 |
| 741 | PNC | TTGCC | 0.0002356 |
| 742 | PNC | TGCCG | 0.0003540 |
| 743 | PNC | TTGCA | 0.0004291 |
| 744 | PNC | TTTCA | 0.0003410 |
| 745 | PNC | TACTT | 0.0002054 |
| 746 | PNC | GGCAT | 0.0002773 |
| 747 | PNC | GTCCA | 0.0002003 |
| 748 | PNC | GTCCC | 0.0004498 |
| 749 | PNC | CTCAG | 0.0002709 |
| 750 | PNC | GTCCT | 0.0003123 |
| 751 | PNC | TACTG | 0.0003350 |
| 752 | PNC | AGCGT | 0.0002828 |
| 753 | PNC | GGCAC | 0.0003544 |
| 754 | PNC | GGCAA | 0.0003264 |
| 755 | PNC | CCGGA | 0.0005487 |
| 756 | PNC | CCGGC | 0.0006171 |
| 757 | PNC | CCGGG | 0.0003918 |
| 758 | PNC | AGAGT | 0.0002553 |
| 759 | PNC | GAGCG | 0.0004959 |
| 760 | PNC | GTTAT | 0.0000791 |
| 761 | PNC | CTCTC | 0.0002555 |
| 762 | PNC | CCGGT | 0.0003343 |
| 763 | PNC | AGCGC | 0.0006157 |
| 764 | PNC | AGGGC | 0.0003207 |
| 765 | PNC | AGCGA | 0.0003669 |
| 766 | PNC | AGCGG | 0.0002425 |
| 767 | PNC | TTTCC | 0.0002414 |
| 768 | PNC | GATTA | 0.0004240 |
| 769 | PNC | TTCGG | 0.0001123 |
| 770 | PNC | GATTC | 0.0003117 |
| 771 | PNC | AGGGA | 0.0002864 |
| 772 | PNC | GATTG | 0.0002295 |
| 773 | PNC | TCAGT | 0.0003309 |
| 774 | PNC | TTTAC | 0.0001951 |
| 775 | PNC | CAGGA | 0.0002803 |
| 776 | PNC | TTTAA | 0.0002847 |
| 777 | PNC | CAGGG | 0.0003553 |
| 778 | PNC | TTTAG | 0.0003010 |
| 779 | PNC | CTTCT | 0.0002379 |
| 780 | PNC | GATTT | 0.0001858 |
| 781 | PNC | CTTCC | 0.0003764 |
| 782 | PNC | CTTCA | 0.0002054 |
| 783 | PNC | TCAGG | 0.0002293 |
| 784 | PNC | CTTCG | 0.0001397 |
| 785 | PNC | TCAGA | 0.0003841 |
| 786 | PNC | TTTAT | 0.0002157 |
| 787 | PNC | TCAGC | 0.0002777 |
| 788 | PNC | GACAT | 0.0001878 |
| 789 | PNC | CTCTT | 0.0003122 |
| 790 | PNC | TTGTT | 0.0002459 |
| 791 | PNC | AAAAT | 0.0004113 |
| 792 | PNC | TCAAA | 0.0002702 |
| 793 | PNC | GACAA | 0.0001646 |
| 794 | PNC | GACAC | 0.0003240 |
| 795 | PNC | GACAG | 0.0002171 |
| 796 | PNC | AAAAA | 0.0003322 |
| 797 | PNC | TTGTG | 0.0004075 |
| 798 | PNC | AAAAC | 0.0001896 |
| 799 | PNC | CTCTA | 0.0002532 |
| 800 | PNC | TTGTC | 0.0002138 |
| 801 | PNC | AAAAG | 0.0002953 |
| 802 | PNC | TTGTA | 0.0002044 |
| 803 | PNC | CATTT | 0.0002765 |
| 804 | PNC | GGATT | 0.0002313 |
| 805 | PNC | CCTCT | 0.0002344 |
| 806 | PNC | CGTTG | 0.0003175 |
| 807 | PNC | CGTTC | 0.0002437 |
| 808 | PNC | CGTTA | 0.0001422 |
| 809 | PNC | GGGGT | 0.0002422 |
| 810 | PNC | CCTCC | 0.0002851 |
| 811 | PNC | CATTG | 0.0003395 |
| 812 | PNC | CCTCA | 0.0002457 |
| 813 | PNC | CATTA | 0.0003003 |
| 814 | PNC | CCTCG | 0.0004557 |
| 815 | PNC | CATTC | 0.0003452 |
| 816 | PNC | GGATA | 0.0001937 |
| 817 | PNC | ACCCG | 0.0001873 |
| 818 | PNC | CGTTT | 0.0001544 |
| 819 | PNC | TTTTT | 0.0003522 |
| 820 | PNC | AGTGA | 0.0002627 |
| 821 | PNC | CGCCG | 0.0022684 |
| 822 | PNC | AGTGC | 0.0003694 |
| 823 | PNC | CGCCA | 0.0004852 |
| 824 | PNC | CGCCC | 0.0014612 |
| 825 | PNC | AGTGG | 0.0003321 |
| 826 | PNC | CAGGC | 0.0002762 |
| 827 | PNC | TTTTG | 0.0002923 |
| 828 | PNC | TTCGT | 0.0001187 |
| 829 | PNC | TTTTA | 0.0002549 |
| 830 | PNC | CTGCA | 0.0004487 |
| 831 | PNC | CGCCT | 0.0007032 |
| 832 | PNC | AGTGT | 0.0003047 |
| 833 | PNC | TGCGC | 0.0013728 |
| 834 | PNC | ATGGG | 0.0002198 |
| 835 | PNC | GCCTA | 0.0004448 |
| 836 | PNC | ATGGC | 0.0002111 |
| 837 | PNC | ATGGA | 0.0003025 |
| 838 | PNC | CCAGC | 0.0003993 |
| 839 | PNC | CCAGA | 0.0002950 |
| 840 | PNC | CCAGG | 0.0003895 |
| 841 | PNC | GAACT | 0.0002603 |
| 842 | PNC | GGGGA | 0.0002486 |
| 843 | PNC | ATGGT | 0.0001414 |
| 844 | PNC | ATCCC | 0.0002536 |
| 845 | PNC | GAACA | 0.0001796 |
| 846 | PNC | GAACC | 0.0005074 |
| 847 | PNC | TCGCT | 0.0007844 |
| 848 | PNC | GTCTC | 0.0002673 |
| 849 | PNC | GAACG | 0.0003053 |
| 850 | PNC | GTGAG | 0.0002318 |
| 851 | PNC | AACCA | 0.0002507 |
| 852 | PNC | AACCC | 0.0002555 |
| 853 | PNC | AACCG | 0.0004662 |
| 854 | PNC | GTTGG | 0.0001688 |
| 855 | PNC | ATCGT | 0.0001356 |
| 856 | PNC | GCCGG | 0.0004165 |
| 857 | PNC | GGGGG | 0.0002455 |
| 858 | PNC | ATCGC | 0.0002698 |
| 859 | PNC | ATCGA | 0.0001418 |
| 860 | PNC | ATCGG | 0.0001852 |
| 861 | PNC | GTAGG | 0.0002134 |
| 862 | PNC | GCCTC | 0.0002123 |
| 863 | PNC | GGGTT | 0.0001982 |
| 864 | PNC | GTAGC | 0.0003096 |
| 865 | PNC | GCCTG | 0.0003344 |
| 866 | PNC | CTAAG | 0.0004866 |
| 867 | PNC | CTAAA | 0.0003576 |
| 868 | PNC | CTAAC | 0.0001778 |
| 869 | PNC | GGGTA | 0.0002594 |
| 870 | PNC | GTAGT | 0.0001700 |
| 871 | PNC | GTCGC | 0.0003812 |
| 872 | PNC | GCCTT | 0.0002265 |
| 873 | PNC | GGGTG | 0.0003087 |
| 874 | PNC | CTAAT | 0.0006286 |
| 875 | PNC | GCACA | 0.0003253 |
| 876 | PNC | GCACG | 0.0001416 |
| 877 | PNC | AGAAG | 0.0002658 |
| 878 | PNC | CCCCC | 0.0003086 |
| 879 | PNC | TTATT | 0.0001934 |
| 880 | PNC | GGCAG | 0.0004141 |
| 881 | PNC | TTATA | 0.0003217 |
| 882 | PNC | CAGGT | 0.0002277 |
| 883 | PNC | TTATG | 0.0001757 |
| 884 | PNC | AGAAA | 0.0004959 |
| 885 | PNC | ACCTT | 0.0003427 |
| 886 | PNC | CAGCT | 0.0002979 |
| 887 | PNC | ACCTC | 0.0003386 |
| 888 | PNC | ACCTA | 0.0002833 |
| 889 | PNC | ACCTG | 0.0003121 |
| 890 | PNC | CAGCG | 0.0003924 |
| 891 | PNC | CAGCC | 0.0003842 |
| 892 | PNC | CAGCA | 0.0005489 |
| 893 | PNC | ACTGT | 0.0002371 |
| 894 | PNC | TCACA | 0.0003113 |
| 895 | PNC | TGAAT | 0.0001845 |
| 896 | PNC | TCACC | 0.0002661 |
| 897 | PNC | ATAAG | 0.0003139 |
| 898 | PNC | TCACG | 0.0001456 |
| 899 | PNC | CTTGG | 0.0002594 |
| 900 | PNC | GAGTC | 0.0009093 |
| 901 | PNC | GAGTA | 0.0001569 |
| 902 | PNC | CTTGC | 0.0003508 |
| 903 | PNC | GAGTG | 0.0002592 |
| 904 | PNC | CTTGA | 0.0002723 |
| 905 | PNC | TGAAG | 0.0001915 |
| 906 | PNC | ACTGG | 0.0002550 |
| 907 | PNC | TCACT | 0.0001983 |
| 908 | PNC | TGAAC | 0.0002799 |
| 909 | PNC | ATAAT | 0.0001322 |
| 910 | PNC | TGAAA | 0.0003585 |
| 911 | PNC | CTTGT | 0.0004873 |
| 912 | PNC | TATGA | 0.0002231 |
| 913 | PNC | GAGTT | 0.0001554 |
| 914 | PNC | TACGA | 0.0000987 |
| 915 | PNC | GCCAC | 0.0003498 |
| 916 | PNC | GCCAA | 0.0002726 |
| 917 | PNC | GCCAG | 0.0002863 |
| 918 | PNC | TTCTG | 0.0003214 |
| 919 | PNC | GCCAT | 0.0002699 |
| 920 | PNC | GGCTC | 0.0002804 |
| 921 | PNC | GATCT | 0.0002197 |
| 922 | PNC | GTGAC | 0.0002495 |
| 923 | PNC | GTGAA | 0.0004937 |
| 924 | PNC | CGCGT | 0.0006778 |
| 925 | PNC | ACGCA | 0.0001528 |
| 926 | PNC | TTAAT | 0.0002200 |
| 927 | PNC | CGCGA | 0.0010164 |
| 928 | PNC | CGCGC | 0.0011794 |
| 929 | PNC | GATCG | 0.0001286 |
| 930 | PNC | AGAAC | 0.0003734 |
| 931 | PNC | GATCA | 0.0002840 |
| 932 | PNC | GTGAT | 0.0001699 |
| 933 | PNC | GATCC | 0.0004067 |
| 934 | PNC | TTAAC | 0.0004953 |
| 935 | PNC | TTAAA | 0.0002325 |
| 936 | PNC | AGTAA | 0.0002551 |
| 937 | PNC | TTAAG | 0.0003359 |
| 938 | PNC | AGGCC | 0.0002646 |
| 939 | PNC | CTGAT | 0.0004301 |
| 940 | PNC | TCGAC | 0.0002150 |
| 941 | PNC | TTCCT | 0.0004163 |
| 942 | PNC | AGCCG | 0.0001896 |
| 943 | PNC | ATGCC | 0.0002457 |
| 944 | PNC | CAGTT | 0.0003291 |
| 945 | PNC | CCACT | 0.0001945 |
| 946 | PNC | AGCCC | 0.0003319 |
| 947 | PNC | ATGCG | 0.0002720 |
| 948 | PNC | AGCCA | 0.0002332 |
| 949 | PNC | TCGAG | 0.0001815 |
| 950 | PNC | TTCCC | 0.0002995 |
| 951 | PNC | CTGAG | 0.0004179 |
| 952 | PNC | TTCCA | 0.0002965 |
| 953 | PNC | TTCCG | 0.0003473 |
| 954 | PNC | CTGAC | 0.0003682 |
| 955 | PNC | CTGAA | 0.0002620 |
| 956 | PNC | CCACG | 0.0002389 |
| 957 | PNC | CAGTG | 0.0002772 |
| 958 | PNC | ACCAG | 0.0003865 |
| 959 | PNC | AGCCT | 0.0003595 |
| 960 | PNC | CCACC | 0.0003259 |
| 961 | PNC | CAGTC | 0.0002570 |
| 962 | PNC | CCACA | 0.0002847 |
| 963 | PNC | CAGTA | 0.0002062 |
| 964 | PNC | TAGAT | 0.0001950 |
| 965 | PNC | ATCAG | 0.0001926 |
| 966 | PNC | CGTCG | 0.0003177 |
| 967 | PNC | ATTTC | 0.0002662 |
| 968 | PNC | CGTCC | 0.0004600 |
| 969 | PNC | CGTCA | 0.0006833 |
| 970 | PNC | TAGAG | 0.0002502 |
| 971 | PNC | GCTTT | 0.0003555 |
| 972 | PNC | TAGAC | 0.0001319 |
| 973 | PNC | TACGT | 0.0002421 |
| 974 | PNC | TAGAA | 0.0002551 |
| 975 | PNC | TATCC | 0.0005139 |
| 976 | PNC | TATCA | 0.0003187 |
| 977 | PNC | ATCAC | 0.0002026 |
| 978 | PNC | TATCG | 0.0001135 |
| 979 | PNC | AAACG | 0.0002301 |
| 980 | PNC | ACGTG | 0.0001168 |
| 981 | PNC | TCCTG | 0.0003749 |
| 982 | PNC | GTACC | 0.0002454 |
| 983 | PNC | TGCCC | 0.0002854 |
| 984 | PNC | TCCTC | 0.0003044 |
| 985 | PNC | GTACG | 0.0002067 |
| 986 | PNC | TCCTA | 0.0001815 |
| 987 | PNC | GGAAG | 0.0003675 |
| 988 | PNC | AAACC | 0.0001962 |
| 989 | PNC | GCGGG | 0.0007650 |
| 990 | PNC | TCCTT | 0.0003161 |
| 991 | PNC | GCGGA | 0.0003710 |
| 992 | PNC | GCGGC | 0.0005448 |
| 993 | PNC | ACGTC | 0.0004201 |
| 994 | PNC | GCAGG | 0.0004496 |
| 995 | PNC | ACCAA | 0.0005359 |
| 996 | PNC | TCTCG | 0.0002295 |
| 997 | PNC | CTACA | 0.0003284 |
| 998 | PNC | GCAGC | 0.0004894 |
| 999 | PNC | AGCTC | 0.0002908 |
| 1000 | PNC | GCAGA | 0.0005021 |
| 1001 | PNC | TGGAC | 0.0002551 |
| 1002 | PNC | ACCAC | 0.0001882 |
| 1003 | PNC | TGGAT | 0.0004328 |
| 1004 | PNC | TCTCT | 0.0002365 |
| 1005 | PNC | AGCTT | 0.0001944 |
| 1006 | PNC | GCAGT | 0.0002796 |
| 1007 | PNC | TCGTT | 0.0001420 |
| 1008 | PNC | CCCAG | 0.0002291 |
| 1009 | PNC | GGCGT | 0.0002602 |
| 1010 | PNC | ACACG | 0.0002543 |
| 1011 | PNC | CGGAT | 0.0001358 |
| 1012 | PNC | ACACA | 0.0006082 |
| 1013 | PNC | CCCAA | 0.0004136 |
| 1014 | PNC | ATATG | 0.0001663 |
| 1015 | PNC | ATATA | 0.0002178 |
| 1016 | PNC | ATATC | 0.0005745 |
| 1017 | PNC | CGGAG | 0.0002691 |
| 1018 | PNC | ACACT | 0.0002538 |
| 1019 | PNC | GAGAG | 0.0002390 |
| 1020 | PNC | CCCAT | 0.0002759 |
| 1021 | PNC | GAGAA | 0.0005459 |
| 1022 | PNC | CGGAA | 0.0005159 |
| 1023 | PNC | GTTGT | 0.0003830 |
| 1024 | PNC | ATATT | 0.0003745 |
| 1025 | PNC | CCATC | 0.0002288 |
| 1026 | PNC | CCATA | 0.0002277 |
| 1027 | PNC | ATTTT | 0.0002540 |
| 1028 | PNC | GGATG | 0.0003039 |
| 1029 | PNC | TGTGT | 0.0003687 |
| 1030 | PNC | GAATG | 0.0002506 |
| 1031 | PNC | ACTCT | 0.0002360 |
| 1032 | PNC | GAATA | 0.0001916 |
| 1033 | PNC | TATCT | 0.0004574 |
| 1034 | PNC | GGGAC | 0.0002462 |
| 1035 | PNC | ATTTA | 0.0002225 |
| 1036 | PNC | GGGAA | 0.0002252 |
| 1037 | PNC | GAGCA | 0.0004151 |
| 1038 | PNC | GGGAG | 0.0002733 |
| 1039 | PNC | ATTTG | 0.0002105 |
| 1040 | PNC | GAATT | 0.0005837 |
| 1041 | PNC | ACTCA | 0.0002404 |
| 1042 | PNC | TGTGG | 0.0002847 |
| 1043 | PNC | ACTCC | 0.0002058 |
| 1044 | PNC | TGTGA | 0.0003817 |
| 1045 | PNC | TGTGC | 0.0003137 |
| 1046 | PNC | GGATC | 0.0002450 |
| 1047 | PNC | TATGG | 0.0001907 |
| 1048 | PNC | TCGAA | 0.0001896 |
| 1049 | PNC | GGCGG | 0.0009986 |
| 1050 | PNC | TATGC | 0.0001735 |
| 1051 | PNC | CGAAG | 0.0002997 |
| 1052 | PNC | GTGTC | 0.0002700 |
| 1053 | PNC | CAATA | 0.0002011 |
| 1054 | PNC | CAATC | 0.0005375 |
| 1055 | PNC | CAATG | 0.0002360 |
| 1056 | PNC | CACCT | 0.0003117 |
| 1057 | PNC | TAAAG | 0.0002340 |
| 1058 | PNC | GATGT | 0.0002029 |
| 1059 | PNC | TAAAA | 0.0002597 |
| 1060 | PNC | TAAAC | 0.0002177 |
| 1061 | PNC | CACCC | 0.0002815 |
| 1062 | PNC | CACCA | 0.0002940 |
| 1063 | PNC | CACCG | 0.0002440 |
| 1064 | PNC | TAAAT | 0.0002446 |
| 1065 | PNC | GATGA | 0.0005695 |
| 1066 | PNC | GATGC | 0.0002482 |
| 1067 | PNC | GATGG | 0.0002181 |
| 1068 | PNC | AACTT | 0.0001881 |
| 1069 | PNC | AAGCG | 0.0003908 |
| 1070 | PNC | CCCTG | 0.0004320 |
| 1071 | PNC | AAGCA | 0.0002272 |
| 1072 | PNC | AAGCC | 0.0001962 |
| 1073 | PNC | GCTAG | 0.0002508 |
| 1074 | PNC | GCTAA | 0.0003895 |
| 1075 | PNC | GAGAT | 0.0004242 |
| 1076 | PNC | CCCTA | 0.0002330 |
| 1077 | PNC | AAGCT | 0.0002528 |
| 1078 | PNC | AACTG | 0.0002867 |
| 1079 | PNC | AACTA | 0.0002326 |
| 1080 | PNC | AACTC | 0.0003622 |
| 1081 | PNC | GCTAT | 0.0001414 |
| 1082 | PNC | GTGTA | 0.0001326 |
| 1083 | PNC | CGTCT | 0.0002036 |
| 1084 | PNC | CGCAA | 0.0013613 |
| 1085 | PNC | ATTAC | 0.0002117 |
| 1086 | PNC | TTCGC | 0.0002868 |
| 1087 | PNC | TTCGA | 0.0002168 |
| 1088 | PNC | CGCGG | 0.0014027 |
| 1089 | PNC | CGTGC | 0.0001225 |
| 1090 | PNC | CGTGA | 0.0001641 |
| 1091 | PNC | CGTGG | 0.0002916 |
| 1092 | PNC | ATTAG | 0.0001718 |
| 1093 | PNC | TGTCA | 0.0003080 |
| 1094 | PNC | TGTCC | 0.0003182 |
| 1095 | PNC | TGTCG | 0.0001875 |
| 1096 | PNC | AGTAT | 0.0001369 |
| 1097 | PNC | CCGTT | 0.0001542 |
| 1098 | PNC | CAAAT | 0.0002784 |
| 1099 | PNC | TACGG | 0.0000935 |
| 1100 | PNC | TGTCT | 0.0002290 |
| 1101 | PNC | TACGC | 0.0001543 |
| 1102 | PNC | CAAAC | 0.0002966 |
| 1103 | PNC | CCGTA | 0.0001084 |
| 1104 | PNC | CAAAA | 0.0002852 |
| 1105 | PNC | CCGTC | 0.0004695 |
| 1106 | PNC | CAAAG | 0.0002747 |
| 1107 | PNC | AGTAC | 0.0001269 |
| 1108 | PNC | ACGTT | 0.0002044 |
| 1109 | PNC | CCGTG | 0.0002461 |
| 1110 | PNC | GCGCA | 0.0006124 |
| 1111 | PNC | AGGCA | 0.0003282 |
| 1112 | PNC | AGGCG | 0.0004446 |
| 1113 | PNC | TCTGT | 0.0003610 |
| 1114 | PNC | GCGCG | 0.0018344 |
| 1115 | PNC | GGAAA | 0.0003587 |
| 1116 | PNC | GGAAC | 0.0002694 |
| 1117 | PNC | CATGG | 0.0003042 |
| 1118 | PNC | CATGA | 0.0001764 |
| 1119 | PNC | GTTGC | 0.0004516 |
| 1120 | PNC | CATGC | 0.0002564 |
| 1121 | PNC | TCTGA | 0.0003078 |
| 1122 | PNC | GTTTC | 0.0002551 |
| 1123 | PNC | TCTGC | 0.0003787 |
| 1124 | PNC | GCGCT | 0.0005317 |
| 1125 | PNC | TCTGG | 0.0003825 |
| 1126 | PNC | AGGCT | 0.0002322 |
| 1127 | PNC | CATGT | 0.0002142 |
| 1128 | PNC | ACCAT | 0.0002828 |
| 1129 | PNC | GGTAA | 0.0002489 |
| 1130 | PNC | GGAAT | 0.0002135 |
| 1131 | PNC | GTTCT | 0.0004099 |
| 1132 | PNC | TTTTC | 0.0002743 |
| 1133 | PNC | CTTTT | 0.0002963 |
| 1134 | PNC | ATTAT | 0.0005464 |
| 1135 | PNC | ACAGA | 0.0002179 |
| 1136 | PNC | ACAGC | 0.0003998 |
| 1137 | PNC | GGAGG | 0.0003495 |
| 1138 | PNC | ACAGG | 0.0002541 |
| 1139 | PNC | GTTCG | 0.0001079 |
| 1140 | PNC | CTTTC | 0.0002049 |
| 1141 | PNC | ATTAA | 0.0002471 |
| 1142 | PNC | CTTTA | 0.0003481 |
| 1143 | PNC | GTTCC | 0.0006081 |
| 1144 | PNC | CTTTG | 0.0002731 |
| 1145 | PNC | GTTCA | 0.0002455 |
| 1146 | PNC | ATAGC | 0.0001144 |
| 1147 | PNC | GGTTC | 0.0004011 |
| 1148 | PNC | ACAGT | 0.0003283 |
| 1149 | PNC | CTCGT | 0.0001835 |
| 1150 | PNC | AATAA | 0.0002241 |
| 1151 | PNC | AATAC | 0.0002450 |
| 1152 | PNC | TTGAT | 0.0002105 |
| 1153 | PNC | AATAG | 0.0002314 |
| 1154 | PNC | TACCC | 0.0001436 |
| 1155 | PNC | TCCCG | 0.0004641 |
| 1156 | PNC | ACGGT | 0.0001132 |
| 1157 | PNC | TCCCC | 0.0004249 |
| 1158 | PNC | TCCCA | 0.0003196 |
| 1159 | PNC | ACGGC | 0.0002149 |
| 1160 | PNC | ACGGA | 0.0001709 |
| 1161 | PNC | CTCGG | 0.0002507 |
| 1162 | PNC | AATAT | 0.0002173 |
| 1163 | PNC | CTCGA | 0.0001775 |
| 1164 | PNC | TTGAG | 0.0002936 |
| 1165 | PNC | CTCGC | 0.0004671 |
| 1166 | PNC | TCCCT | 0.0003112 |
| 1167 | PNC | GCGGT | 0.0004339 |
| 1168 | PNC | GTCAG | 0.0004793 |
| 1169 | PNC | GTCAC | 0.0002338 |
| 1170 | PNC | GTCAA | 0.0003340 |
| 1171 | PNC | TGATA | 0.0003648 |
| 1172 | PNC | TGATC | 0.0002300 |
| 1173 | PNC | TGATG | 0.0002032 |
| 1174 | PNC | CCGAT | 0.0000931 |
| 1175 | PNC | GGCCA | 0.0002865 |
| 1176 | PNC | GTCAT | 0.0002547 |
| 1177 | PNC | GGCCG | 0.0006871 |
| 1178 | PNC | AATTT | 0.0002105 |
| 1179 | PNC | CCGAC | 0.0002197 |
| 1180 | PNC | CGGGA | 0.0002361 |
| 1181 | PNC | CCGAA | 0.0001836 |
| 1182 | PNC | CCGAG | 0.0002676 |
| 1183 | PNC | TGATT | 0.0002627 |
| 1184 | PNC | TTGAA | 0.0002007 |
| 1185 | PNC | GTTTT | 0.0003629 |
| 1186 | PNC | CTCAT | 0.0002273 |
| 1187 | PNC | CGTGT | 0.0001909 |
| 1188 | PNC | TCGGT | 0.0001074 |
| 1189 | PNC | TTGAC | 0.0002519 |
| 1190 | PNC | GCCGT | 0.0004188 |
| 1191 | PNC | GTTTG | 0.0002229 |
| 1192 | PNC | GTACT | 0.0002019 |
| 1193 | PNC | GTCGG | 0.0002012 |
| 1194 | PNC | TCGGG | 0.0003552 |
| 1195 | PNC | ACGTA | 0.0003887 |
| 1196 | PNC | TCGGC | 0.0004894 |
| 1197 | PNC | TCGGA | 0.0001462 |
| 1198 | PNC | CGACC | 0.0001888 |
| 1199 | PNC | CGACA | 0.0001289 |
| 1200 | PNC | CGACG | 0.0002043 |
| 1201 | PNC | CACAT | 0.0003421 |
| 1202 | PNC | AAGGA | 0.0002805 |
| 1203 | PNC | CTTAT | 0.0003320 |
| 1204 | PNC | AAGGC | 0.0003342 |
| 1205 | PNC | TTTGG | 0.0002784 |
| 1206 | PNC | TTTGA | 0.0002157 |
| 1207 | PNC | AAGGG | 0.0002853 |
| 1208 | PNC | TTTGC | 0.0002285 |
| 1209 | PNC | TACCG | 0.0002862 |
| 1210 | PNC | CGACT | 0.0005245 |
| 1211 | PNC | TTTGT | 0.0002288 |
| 1212 | PNC | CTTAG | 0.0003155 |
| 1213 | PNC | CTTAA | 0.0002790 |
| 1214 | PNC | AAGGT | 0.0002544 |
| 1215 | PNC | CTTAC | 0.0001531 |
| 1216 | PNC | AATTC | 0.0003804 |
| 1217 | PNC | CAGAA | 0.0003151 |
| 1218 | PNC | AATTA | 0.0002888 |
| 1219 | PNC | GGTAT | 0.0001336 |
| 1220 | PNC | AAAGT | 0.0002658 |
| 1221 | PNC | GACGC | 0.0003991 |
| 1222 | PNC | GACGA | 0.0001832 |
| 1223 | PNC | GACGG | 0.0002775 |
| 1224 | PNC | GGTTA | 0.0001299 |
| 1225 | PNC | GGTAG | 0.0001419 |
| 1226 | PNC | AAAGC | 0.0002453 |
| 1227 | PNC | TCGTC | 0.0002031 |
| 1228 | PNC | AAAGA | 0.0002769 |
| 1229 | PNC | GGTAC | 0.0002317 |
| 1230 | PNC | AAAGG | 0.0004978 |
| 1231 | PNC | AGCTG | 0.0003244 |
| 1232 | PNC | GGTTG | 0.0002003 |
| 1233 | PNC | TCGTA | 0.0001113 |
| 1234 | PNC | GACGT | 0.0003009 |
| 1235 | PNC | CTATG | 0.0001634 |
| 1236 | PNC | TGCAA | 0.0003175 |
| 1237 | PNC | TGGAG | 0.0002700 |
| 1238 | PNC | CTATC | 0.0004384 |
| 1239 | PNC | CAGAG | 0.0002826 |
| 1240 | PNC | GCATT | 0.0004370 |
| 1241 | PNC | TGCAG | 0.0003770 |
| 1242 | PNC | TAGTA | 0.0001241 |
| 1243 | PNC | TCTCA | 0.0002657 |
| 1244 | PNC | TAGTC | 0.0001844 |
| 1245 | PNC | TTTCG | 0.0001510 |
| 1246 | PNC | TACCT | 0.0003058 |
| 1247 | PNC | TAGTG | 0.0002429 |
| 1248 | PNC | TGGAA | 0.0006052 |
| 1249 | PNC | GCATC | 0.0002791 |
| 1250 | PNC | GCATA | 0.0002298 |
| 1251 | PNC | TGCAT | 0.0003791 |
| 1252 | PNC | TCTCC | 0.0002490 |
| 1253 | PNC | TACCA | 0.0001480 |
| 1254 | PNC | AGCTA | 0.0003064 |
| 1255 | PNC | TAGTT | 0.0001448 |
| 1256 | PNC | GGCCT | 0.0003238 |
| 1257 | PNC | GGGTC | 0.0002147 |
| 1258 | PNC | CCCGT | 0.0002321 |
| 1259 | PNC | TACTC | 0.0002445 |
| 1260 | PNC | AGGGT | 0.0002946 |
| 1261 | PNC | CATCT | 0.0002477 |
| 1262 | PNC | AGAGC | 0.0003572 |
| 1263 | PNC | GGCGA | 0.0003680 |
| 1264 | PNC | CACAG | 0.0003939 |
| 1265 | PNC | ATCAT | 0.0003605 |
| 1266 | PNC | AGGGG | 0.0003328 |
| 1267 | PNC | CATCA | 0.0002023 |
| 1268 | PNC | CCCGG | 0.0003469 |
| 1269 | PNC | CATCC | 0.0002286 |
| 1270 | PNC | CCCGA | 0.0003448 |
| 1271 | PNC | CCCGC | 0.0005019 |
| 1272 | PNC | CATCG | 0.0002409 |
| 1273 | PNC | CCAGT | 0.0003338 |
| 1274 | PNC | CACGG | 0.0003765 |
| 1275 | PNC | CACGC | 0.0001984 |
| 1276 | PNC | CACGA | 0.0003097 |
| 1277 | PNC | CCTGC | 0.0002509 |
| 1278 | PNC | CACGT | 0.0001434 |
| 1279 | PNC | CAATT | 0.0002204 |
| 1280 | PNC | CCATG | 0.0003038 |
| 1281 | PNC | GTGTT | 0.0002355 |
| 1282 | PNC | TCCGT | 0.0001105 |
| 1283 | PNC | CTGTA | 0.0001929 |
| 1284 | PNC | TTCTC | 0.0002732 |
| 1285 | PNC | CTGTC | 0.0003181 |
| 1286 | PNC | TTCTA | 0.0002088 |
| 1287 | PNC | CTCCT | 0.0002804 |
| 1288 | PNC | GGCCC | 0.0002368 |
| 1289 | PNC | CTGTG | 0.0003562 |
| 1290 | PNC | CGATG | 0.0001284 |
| 1291 | PNC | TCCGC | 0.0006547 |
| 1292 | PNC | TCCGA | 0.0002466 |
| 1293 | PNC | TCCGG | 0.0008677 |
| 1294 | PNC | CTCTG | 0.0002987 |
| 1295 | PNC | CTCCA | 0.0003025 |
| 1296 | PNC | CTCCC | 0.0003321 |
| 1297 | PNC | CTGTT | 0.0002074 |
| 1298 | PNC | TTCTT | 0.0002089 |
| 1299 | PNC | CTCCG | 0.0003143 |
| 1300 | PNC | TGCTC | 0.0004453 |
| 1301 | PNC | CTACG | 0.0002151 |
| 1302 | PNC | TGCTA | 0.0002932 |
| 1303 | PNC | TGCTG | 0.0003451 |
| 1304 | PNC | CTACC | 0.0001595 |
| 1305 | PNC | AAACA | 0.0002168 |
| 1306 | PNC | ACATA | 0.0002760 |
| 1307 | PNC | GCAAT | 0.0002319 |
| 1308 | PNC | CCCCG | 0.0004787 |
| 1309 | PNC | GTTGA | 0.0002909 |
| 1310 | PNC | TCAAG | 0.0002865 |
| 1311 | PNC | CTACT | 0.0001520 |
| 1312 | PNC | AAACT | 0.0002793 |
| 1313 | PNC | TGCTT | 0.0002551 |
| 1314 | PNC | CCCAC | 0.0002273 |
| 1315 | PNC | GCAAG | 0.0003996 |
| 1316 | PNC | GCAAA | 0.0003871 |
| 1317 | PNC | GCAAC | 0.0003005 |
| 1318 | PNC | ACGGG | 0.0001831 |
| 1319 | PNC | AGTAG | 0.0001942 |
| 1320 | PNC | ACACC | 0.0008425 |
| 1321 | PNC | GCTCA | 0.0005502 |
| 1322 | PNC | GCGCC | 0.0009348 |
| 1323 | PNC | TCGAT | 0.0002723 |
| 1324 | PNC | CCATT | 0.0002075 |
| 1325 | PNC | AACCT | 0.0004583 |
| 1326 | PNC | CGGGG | 0.0003843 |
| 1327 | PNC | CGAGT | 0.0004867 |
| 1328 | PNC | GGCTT | 0.0002717 |
| 1329 | PNC | CAGAT | 0.0002679 |
| 1330 | PNC | CGGGC | 0.0006325 |
| 1331 | PNC | GGTTT | 0.0002506 |
| 1332 | PNC | CGCTA | 0.0015140 |
| 1333 | PNC | ATGCA | 0.0004774 |
| 1334 | PNC | CGCTC | 0.0006478 |
| 1335 | PNC | TCAAT | 0.0001638 |
| 1336 | PNC | CGCTG | 0.0003304 |
| 1337 | PNC | CGAGG | 0.0003594 |
| 1338 | PNC | CGGGT | 0.0001750 |
| 1339 | PNC | CAGAC | 0.0003818 |
| 1340 | PNC | AGTCG | 0.0002118 |
| 1341 | PNC | CGAGC | 0.0002032 |
| 1342 | PNC | CGAGA | 0.0002265 |
| 1343 | PNC | TCGTG | 0.0001216 |
| 1344 | PNC | TCAAC | 0.0004861 |
| 1345 | PNC | TCTTG | 0.0002943 |
| 1346 | PNC | CGCTT | 0.0001799 |
| 1347 | PNC | TCCAT | 0.0002116 |
| 1348 | PNC | ATAGG | 0.0002814 |
| 1349 | PNC | CCAAC | 0.0002655 |
| 1350 | PNC | ATAGA | 0.0002937 |
| 1351 | PNC | TATTT | 0.0002865 |
| 1352 | PNC | ATCAA | 0.0001687 |
| 1353 | PNC | GACCT | 0.0003216 |
| 1354 | PNC | GTACA | 0.0001943 |
| 1355 | PNC | ATAGT | 0.0001299 |
| 1356 | PNC | TATTC | 0.0000964 |
| 1357 | PNC | TATTA | 0.0001695 |
| 1358 | PNC | TATTG | 0.0002302 |
| 1359 | PNC | CATAC | 0.0004574 |
| 1360 | PNC | GACCG | 0.0003067 |
| 1361 | PNC | CGGAC | 0.0002030 |
| 1362 | PNC | AGAGG | 0.0002101 |
| 1363 | PNC | GACCC | 0.0002366 |
| 1364 | PNC | GACCA | 0.0004591 |
| 1365 | DPCP_P1 | AA | 0.0002689 |
| 1366 | DPCP_P1 | AC | 0.0004412 |
| 1367 | DPCP_P1 | GT | 0.0002735 |
| 1368 | DPCP_P1 | AG | 0.0003460 |
| 1369 | DPCP_P1 | CC | 0.0002757 |
| 1370 | DPCP_P1 | TT | 0.0003507 |
| 1371 | DPCP_P1 | CG | 0.0002728 |
| 1372 | DPCP_P1 | GG | 0.0002441 |
| 1373 | DPCP_P1 | GC | 0.0002859 |
| 1374 | DPCP_P1 | AT | 0.0003169 |
| 1375 | DPCP_P1 | GA | 0.0003982 |
| 1376 | DPCP_P1 | TG | 0.0005366 |
| 1377 | DPCP_P1 | CT | 0.0002990 |
| 1378 | DPCP_P1 | CA | 0.0005264 |
| 1379 | DPCP_P1 | TC | 0.0000000 |
| 1380 | DPCP_P1 | TA | 0.0000000 |
| 1381 | DPCP_P2 | AA | 0.0006426 |
| 1382 | DPCP_P2 | AC | 0.0004064 |
| 1383 | DPCP_P2 | GT | 0.0003209 |
| 1384 | DPCP_P2 | AG | 0.0004899 |
| 1385 | DPCP_P2 | CC | 0.0010187 |
| 1386 | DPCP_P2 | TT | 0.0003510 |
| 1387 | DPCP_P2 | CG | 0.0002939 |
| 1388 | DPCP_P2 | GG | 0.0006677 |
| 1389 | DPCP_P2 | GC | 0.0034021 |
| 1390 | DPCP_P2 | AT | 0.0003001 |
| 1391 | DPCP_P2 | GA | 0.0003034 |
| 1392 | DPCP_P2 | TG | 0.0003528 |
| 1393 | DPCP_P2 | CT | 0.0002921 |
| 1394 | DPCP_P2 | CA | 0.0004590 |
| 1395 | DPCP_P2 | TC | 0.0010798 |
| 1396 | DPCP_P2 | TA | 0.0003422 |
| 1397 | DPCP_P3 | AA | 0.0002812 |
| 1398 | DPCP_P3 | AC | 0.0003121 |
| 1399 | DPCP_P3 | GT | 0.0029414 |
| 1400 | DPCP_P3 | AG | 0.0002591 |
| 1401 | DPCP_P3 | CC | 0.0003254 |
| 1402 | DPCP_P3 | TT | 0.0002722 |
| 1403 | DPCP_P3 | CG | 0.0004873 |
| 1404 | DPCP_P3 | GG | 0.0002651 |
| 1405 | DPCP_P3 | GC | 0.0003568 |
| 1406 | DPCP_P3 | AT | 0.0004166 |
| 1407 | DPCP_P3 | GA | 0.0003035 |
| 1408 | DPCP_P3 | TG | 0.0002800 |
| 1409 | DPCP_P3 | CT | 0.0012019 |
| 1410 | DPCP_P3 | CA | 0.0005287 |
| 1411 | DPCP_P3 | TC | 0.0003315 |
| 1412 | DPCP_P3 | TA | 0.0002890 |
| 1413 | DPCP_P4 | AA | 0.0003084 |
| 1414 | DPCP_P4 | AC | 0.0003225 |
| 1415 | DPCP_P4 | GT | 0.0004767 |
| 1416 | DPCP_P4 | AG | 0.0002875 |
| 1417 | DPCP_P4 | CC | 0.0003224 |
| 1418 | DPCP_P4 | TT | 0.0002969 |
| 1419 | DPCP_P4 | CG | 0.0066772 |
| 1420 | DPCP_P4 | GG | 0.0003438 |
| 1421 | DPCP_P4 | GC | 0.0003110 |
| 1422 | DPCP_P4 | AT | 0.0003278 |
| 1423 | DPCP_P4 | GA | 0.0002687 |
| 1424 | DPCP_P4 | TG | 0.0004291 |
| 1425 | DPCP_P4 | CT | 0.0002796 |
| 1426 | DPCP_P4 | CA | 0.0008152 |
| 1427 | DPCP_P4 | TC | 0.0003174 |
| 1428 | DPCP_P4 | TA | 0.0053278 |
| 1429 | DPCP_P5 | AA | 0.0002667 |
| 1430 | DPCP_P5 | AC | 0.0005263 |
| 1431 | DPCP_P5 | GT | 0.0002751 |
| 1432 | DPCP_P5 | AG | 0.0000000 |
| 1433 | DPCP_P5 | CC | 0.0002807 |
| 1434 | DPCP_P5 | TT | 0.0003426 |
| 1435 | DPCP_P5 | CG | 0.0002633 |
| 1436 | DPCP_P5 | GG | 0.0002433 |
| 1437 | DPCP_P5 | GC | 0.0002871 |
| 1438 | DPCP_P5 | AT | 0.0003185 |
| 1439 | DPCP_P5 | GA | 0.0003966 |
| 1440 | DPCP_P5 | TG | 0.0005400 |
| 1441 | DPCP_P5 | CT | 0.0002975 |
| 1442 | DPCP_P5 | CA | 0.0004708 |
| 1443 | DPCP_P5 | TC | 0.0003540 |
| 1444 | DPCP_P5 | TA | 0.0006242 |
| 1445 | DPCP_P6 | AA | 0.0006399 |
| 1446 | DPCP_P6 | AC | 0.0004039 |
| 1447 | DPCP_P6 | GT | 0.0003176 |
| 1448 | DPCP_P6 | AG | 0.0004919 |
| 1449 | DPCP_P6 | CC | 0.0007006 |
| 1450 | DPCP_P6 | TT | 0.0003514 |
| 1451 | DPCP_P6 | CG | 0.0002983 |
| 1452 | DPCP_P6 | GG | 0.0006679 |
| 1453 | DPCP_P6 | GC | 0.0021429 |
| 1454 | DPCP_P6 | AT | 0.0003037 |
| 1455 | DPCP_P6 | GA | 0.0003027 |
| 1456 | DPCP_P6 | TG | 0.0003524 |
| 1457 | DPCP_P6 | CT | 0.0002923 |
| 1458 | DPCP_P6 | CA | 0.0004214 |
| 1459 | DPCP_P6 | TC | 0.0014789 |
| 1460 | DPCP_P6 | TA | 0.0003441 |
| 1461 | TPCP_P1 | ACC | 0.0002819 |
| 1462 | TPCP_P1 | ATG | 0.0003121 |
| 1463 | TPCP_P1 | AAG | 0.0036449 |
| 1464 | TPCP_P1 | AAA | 0.0000000 |
| 1465 | TPCP_P1 | ATC | 0.0003267 |
| 1466 | TPCP_P1 | AAC | 0.0002752 |
| 1467 | TPCP_P1 | ATA | 0.0004915 |
| 1468 | TPCP_P1 | AGG | 0.0002619 |
| 1469 | TPCP_P1 | CCT | 0.0003581 |
| 1470 | TPCP_P1 | CTC | 0.0004718 |
| 1471 | TPCP_P1 | AGC | 0.0003023 |
| 1472 | TPCP_P1 | ACA | 0.0002816 |
| 1473 | TPCP_P1 | AGA | 0.0008973 |
| 1474 | TPCP_P1 | CAT | 0.0005400 |
| 1475 | TPCP_P1 | AAT | 0.0003298 |
| 1476 | TPCP_P1 | ATT | 0.0002908 |
| 1477 | TPCP_P1 | CTG | 0.0003101 |
| 1478 | TPCP_P1 | CTA | 0.0003173 |
| 1479 | TPCP_P1 | ACT | 0.0004803 |
| 1480 | TPCP_P1 | CAC | 0.0002895 |
| 1481 | TPCP_P1 | ACG | 0.0003242 |
| 1482 | TPCP_P1 | CAA | 0.0002960 |
| 1483 | TPCP_P1 | AGT | 0.0075445 |
| 1484 | TPCP_P1 | CAG | 0.0003446 |
| 1485 | TPCP_P1 | CCG | 0.0003102 |
| 1486 | TPCP_P1 | CCC | 0.0003294 |
| 1487 | TPCP_P1 | CTT | 0.0002681 |
| 1488 | TPCP_P1 | TAT | 0.0004317 |
| 1489 | TPCP_P1 | GGT | 0.0002845 |
| 1490 | TPCP_P1 | TGT | 0.0009233 |
| 1491 | TPCP_P1 | CGA | 0.0003092 |
| 1492 | TPCP_P1 | CCA | 0.0039289 |
| 1493 | TPCP_P1 | TCT | 0.0002651 |
| 1494 | TPCP_P1 | GAT | 0.0004631 |
| 1495 | TPCP_P1 | CGG | 0.0002731 |
| 1496 | TPCP_P1 | TTT | 0.0000000 |
| 1497 | TPCP_P1 | TGC | 0.0002787 |
| 1498 | TPCP_P1 | GGG | 0.0003457 |
| 1499 | TPCP_P1 | TAG | 0.0000000 |
| 1500 | TPCP_P1 | GGA | 0.0002456 |
| 1501 | TPCP_P1 | TAA | 0.0002853 |
| 1502 | TPCP_P1 | GGC | 0.0003177 |
| 1503 | TPCP_P1 | TAC | 0.0003934 |
| 1504 | TPCP_P1 | TTC | 0.0005371 |
| 1505 | TPCP_P1 | TCG | 0.0003000 |
| 1506 | TPCP_P1 | TTA | 0.0004624 |
| 1507 | TPCP_P1 | TTG | 0.0000000 |
| 1508 | TPCP_P1 | TCC | 0.0000000 |
| 1509 | TPCP_P1 | GAA | 0.0006387 |
| 1510 | TPCP_P1 | TGG | 0.0004056 |
| 1511 | TPCP_P1 | GCA | 0.0003194 |
| 1512 | TPCP_P1 | GTA | 0.0004902 |
| 1513 | TPCP_P1 | GCC | 0.0008590 |
| 1514 | TPCP_P1 | GTC | 0.0003510 |
| 1515 | TPCP_P1 | GCG | 0.0002916 |
| 1516 | TPCP_P1 | GTG | 0.0006661 |
| 1517 | TPCP_P1 | GAG | 0.0034053 |
| 1518 | TPCP_P1 | GTT | 0.0002999 |
| 1519 | TPCP_P1 | GCT | 0.0003023 |
| 1520 | TPCP_P1 | TGA | 0.0000000 |
| 1521 | TPCP_P1 | GAC | 0.0002895 |
| 1522 | TPCP_P1 | CGT | 0.0004066 |
| 1523 | TPCP_P1 | TCA | 0.0013972 |
| 1524 | TPCP_P1 | CGC | 0.0003470 |
| 1525 | TPCP_P2 | ACC | 0.0002801 |
| 1526 | TPCP_P2 | ATG | 0.0003192 |
| 1527 | TPCP_P2 | AAG | 0.0037931 |
| 1528 | TPCP_P2 | AAA | 0.0000000 |
| 1529 | TPCP_P2 | ATC | 0.0003251 |
| 1530 | TPCP_P2 | AAC | 0.0002715 |
| 1531 | TPCP_P2 | ATA | 0.0004854 |
| 1532 | TPCP_P2 | AGG | 0.0002635 |
| 1533 | TPCP_P2 | CCT | 0.0000000 |
| 1534 | TPCP_P2 | CTC | 0.0005018 |
| 1535 | TPCP_P2 | AGC | 0.0003045 |
| 1536 | TPCP_P2 | ACA | 0.0002841 |
| 1537 | TPCP_P2 | AGA | 0.0013088 |
| 1538 | TPCP_P2 | CAT | 0.0000000 |
| 1539 | TPCP_P2 | AAT | 0.0003306 |
| 1540 | TPCP_P2 | ATT | 0.0002890 |
| 1541 | TPCP_P2 | CTG | 0.0003062 |
| 1542 | TPCP_P2 | CTA | 0.0003211 |
| 1543 | TPCP_P2 | ACT | 0.0004784 |
| 1544 | TPCP_P2 | CAC | 0.0002886 |
| 1545 | TPCP_P2 | ACG | 0.0003339 |
| 1546 | TPCP_P2 | CAA | 0.0002975 |
| 1547 | TPCP_P2 | AGT | 0.0053354 |
| 1548 | TPCP_P2 | CAG | 0.0003451 |
| 1549 | TPCP_P2 | CCG | 0.0003116 |
| 1550 | TPCP_P2 | CCC | 0.0003284 |
| 1551 | TPCP_P2 | CTT | 0.0002702 |
| 1552 | TPCP_P2 | TAT | 0.0004082 |
| 1553 | TPCP_P2 | GGT | 0.0002847 |
| 1554 | TPCP_P2 | TGT | 0.0009298 |
| 1555 | TPCP_P2 | CGA | 0.0003075 |
| 1556 | TPCP_P2 | CCA | 0.0046123 |
| 1557 | TPCP_P2 | TCT | 0.0002677 |
| 1558 | TPCP_P2 | GAT | 0.0004292 |
| 1559 | TPCP_P2 | CGG | 0.0002720 |
| 1560 | TPCP_P2 | TTT | 0.0000000 |
| 1561 | TPCP_P2 | TGC | 0.0002770 |
| 1562 | TPCP_P2 | GGG | 0.0003437 |
| 1563 | TPCP_P2 | TAG | 0.0002909 |
| 1564 | TPCP_P2 | GGA | 0.0002426 |
| 1565 | TPCP_P2 | TAA | 0.0002850 |
| 1566 | TPCP_P2 | GGC | 0.0003161 |
| 1567 | TPCP_P2 | TAC | 0.0003948 |
| 1568 | TPCP_P2 | TTC | 0.0005568 |
| 1569 | TPCP_P2 | TCG | 0.0003021 |
| 1570 | TPCP_P2 | TTA | 0.0004412 |
| 1571 | TPCP_P2 | TTG | 0.0003637 |
| 1572 | TPCP_P2 | TCC | 0.0005939 |
| 1573 | TPCP_P2 | GAA | 0.0006401 |
| 1574 | TPCP_P2 | TGG | 0.0004061 |
| 1575 | TPCP_P2 | GCA | 0.0003212 |
| 1576 | TPCP_P2 | GTA | 0.0004905 |
| 1577 | TPCP_P2 | GCC | 0.0006637 |
| 1578 | TPCP_P2 | GTC | 0.0003521 |
| 1579 | TPCP_P2 | GCG | 0.0002925 |
| 1580 | TPCP_P2 | GTG | 0.0006712 |
| 1581 | TPCP_P2 | GAG | 0.0024602 |
| 1582 | TPCP_P2 | GTT | 0.0003056 |
| 1583 | TPCP_P2 | GCT | 0.0003109 |
| 1584 | TPCP_P2 | TGA | 0.0003482 |
| 1585 | TPCP_P2 | GAC | 0.0002918 |
| 1586 | TPCP_P2 | CGT | 0.0004254 |
| 1587 | TPCP_P2 | TCA | 0.0010063 |
| 1588 | TPCP_P2 | CGC | 0.0003460 |
| 1589 | TPCP_P3 | ACC | 0.0002852 |
| 1590 | TPCP_P3 | ATG | 0.0003143 |
| 1591 | TPCP_P3 | AAG | 0.0030537 |
| 1592 | TPCP_P3 | AAA | 0.0000000 |
| 1593 | TPCP_P3 | ATC | 0.0003244 |
| 1594 | TPCP_P3 | AAC | 0.0002705 |
| 1595 | TPCP_P3 | ATA | 0.0004821 |
| 1596 | TPCP_P3 | AGG | 0.0002639 |
| 1597 | TPCP_P3 | CCT | 0.0003607 |
| 1598 | TPCP_P3 | CTC | 0.0003865 |
| 1599 | TPCP_P3 | AGC | 0.0003044 |
| 1600 | TPCP_P3 | ACA | 0.0002829 |
| 1601 | TPCP_P3 | AGA | 0.0013340 |
| 1602 | TPCP_P3 | CAT | 0.0005279 |
| 1603 | TPCP_P3 | AAT | 0.0003340 |
| 1604 | TPCP_P3 | ATT | 0.0002871 |
| 1605 | TPCP_P3 | CTG | 0.0003081 |
| 1606 | TPCP_P3 | CTA | 0.0003204 |
| 1607 | TPCP_P3 | ACT | 0.0004796 |
| 1608 | TPCP_P3 | CAC | 0.0002883 |
| 1609 | TPCP_P3 | ACG | 0.0004056 |
| 1610 | TPCP_P3 | CAA | 0.0002976 |
| 1611 | TPCP_P3 | AGT | 0.0046412 |
| 1612 | TPCP_P3 | CAG | 0.0003429 |
| 1613 | TPCP_P3 | CCG | 0.0003103 |
| 1614 | TPCP_P3 | CCC | 0.0003268 |
| 1615 | TPCP_P3 | CTT | 0.0002678 |
| 1616 | TPCP_P3 | TAT | 0.0003912 |
| 1617 | TPCP_P3 | GGT | 0.0002820 |
| 1618 | TPCP_P3 | TGT | 0.0009276 |
| 1619 | TPCP_P3 | CGA | 0.0003160 |
| 1620 | TPCP_P3 | CCA | 0.0037493 |
| 1621 | TPCP_P3 | TCT | 0.0002665 |
| 1622 | TPCP_P3 | GAT | 0.0004576 |
| 1623 | TPCP_P3 | CGG | 0.0002735 |
| 1624 | TPCP_P3 | TTT | 0.0000000 |
| 1625 | TPCP_P3 | TGC | 0.0002793 |
| 1626 | TPCP_P3 | GGG | 0.0003446 |
| 1627 | TPCP_P3 | TAG | 0.0002700 |
| 1628 | TPCP_P3 | GGA | 0.0002459 |
| 1629 | TPCP_P3 | TAA | 0.0002851 |
| 1630 | TPCP_P3 | GGC | 0.0003164 |
| 1631 | TPCP_P3 | TAC | 0.0003936 |
| 1632 | TPCP_P3 | TTC | 0.0005490 |
| 1633 | TPCP_P3 | TCG | 0.0002964 |
| 1634 | TPCP_P3 | TTA | 0.0004435 |
| 1635 | TPCP_P3 | TTG | 0.0003582 |
| 1636 | TPCP_P3 | TCC | 0.0006037 |
| 1637 | TPCP_P3 | GAA | 0.0006386 |
| 1638 | TPCP_P3 | TGG | 0.0004061 |
| 1639 | TPCP_P3 | GCA | 0.0003176 |
| 1640 | TPCP_P3 | GTA | 0.0004910 |
| 1641 | TPCP_P3 | GCC | 0.0006311 |
| 1642 | TPCP_P3 | GTC | 0.0003538 |
| 1643 | TPCP_P3 | GCG | 0.0002945 |
| 1644 | TPCP_P3 | GTG | 0.0006606 |
| 1645 | TPCP_P3 | GAG | 0.0027278 |
| 1646 | TPCP_P3 | GTT | 0.0003007 |
| 1647 | TPCP_P3 | GCT | 0.0003003 |
| 1648 | TPCP_P3 | TGA | 0.0003581 |
| 1649 | TPCP_P3 | GAC | 0.0002898 |
| 1650 | TPCP_P3 | CGT | 0.0004032 |
| 1651 | TPCP_P3 | TCA | 0.0013987 |
| 1652 | TPCP_P3 | CGC | 0.0003443 |
| 1653 | TPCP_P4 | ACC | 0.0002797 |
| 1654 | TPCP_P4 | ATG | 0.0003163 |
| 1655 | TPCP_P4 | AAG | 0.0036716 |
| 1656 | TPCP_P4 | AAA | 0.0000000 |
| 1657 | TPCP_P4 | ATC | 0.0003253 |
| 1658 | TPCP_P4 | AAC | 0.0002735 |
| 1659 | TPCP_P4 | ATA | 0.0004856 |
| 1660 | TPCP_P4 | AGG | 0.0002653 |
| 1661 | TPCP_P4 | CCT | 0.0003604 |
| 1662 | TPCP_P4 | CTC | 0.0004304 |
| 1663 | TPCP_P4 | AGC | 0.0003021 |
| 1664 | TPCP_P4 | ACA | 0.0002808 |
| 1665 | TPCP_P4 | AGA | 0.0015335 |
| 1666 | TPCP_P4 | CAT | 0.0005236 |
| 1667 | TPCP_P4 | AAT | 0.0003313 |
| 1668 | TPCP_P4 | ATT | 0.0002900 |
| 1669 | TPCP_P4 | CTG | 0.0003100 |
| 1670 | TPCP_P4 | CTA | 0.0003195 |
| 1671 | TPCP_P4 | ACT | 0.0004834 |
| 1672 | TPCP_P4 | CAC | 0.0002884 |
| 1673 | TPCP_P4 | ACG | 0.0003906 |
| 1674 | TPCP_P4 | CAA | 0.0002956 |
| 1675 | TPCP_P4 | AGT | 0.0058674 |
| 1676 | TPCP_P4 | CAG | 0.0003422 |
| 1677 | TPCP_P4 | CCG | 0.0003114 |
| 1678 | TPCP_P4 | CCC | 0.0003286 |
| 1679 | TPCP_P4 | CTT | 0.0002652 |
| 1680 | TPCP_P4 | TAT | 0.0003797 |
| 1681 | TPCP_P4 | GGT | 0.0002790 |
| 1682 | TPCP_P4 | TGT | 0.0009311 |
| 1683 | TPCP_P4 | CGA | 0.0003330 |
| 1684 | TPCP_P4 | CCA | 0.0056042 |
| 1685 | TPCP_P4 | TCT | 0.0002666 |
| 1686 | TPCP_P4 | GAT | 0.0004334 |
| 1687 | TPCP_P4 | CGG | 0.0002733 |
| 1688 | TPCP_P4 | TTT | 0.0000000 |
| 1689 | TPCP_P4 | TGC | 0.0002748 |
| 1690 | TPCP_P4 | GGG | 0.0003426 |
| 1691 | TPCP_P4 | TAG | 0.0002689 |
| 1692 | TPCP_P4 | GGA | 0.0002442 |
| 1693 | TPCP_P4 | TAA | 0.0002859 |
| 1694 | TPCP_P4 | GGC | 0.0003164 |
| 1695 | TPCP_P4 | TAC | 0.0003940 |
| 1696 | TPCP_P4 | TTC | 0.0005292 |
| 1697 | TPCP_P4 | TCG | 0.0003031 |
| 1698 | TPCP_P4 | TTA | 0.0004354 |
| 1699 | TPCP_P4 | TTG | 0.0003595 |
| 1700 | TPCP_P4 | TCC | 0.0005982 |
| 1701 | TPCP_P4 | GAA | 0.0006334 |
| 1702 | TPCP_P4 | TGG | 0.0004056 |
| 1703 | TPCP_P4 | GCA | 0.0003177 |
| 1704 | TPCP_P4 | GTA | 0.0004900 |
| 1705 | TPCP_P4 | GCC | 0.0009655 |
| 1706 | TPCP_P4 | GTC | 0.0003523 |
| 1707 | TPCP_P4 | GCG | 0.0002949 |
| 1708 | TPCP_P4 | GTG | 0.0006629 |
| 1709 | TPCP_P4 | GAG | 0.0032608 |
| 1710 | TPCP_P4 | GTT | 0.0003031 |
| 1711 | TPCP_P4 | GCT | 0.0003062 |
| 1712 | TPCP_P4 | TGA | 0.0003499 |
| 1713 | TPCP_P4 | GAC | 0.0002900 |
| 1714 | TPCP_P4 | CGT | 0.0004224 |
| 1715 | TPCP_P4 | TCA | 0.0011474 |
| 1716 | TPCP_P4 | CGC | 0.0003489 |
| 1717 | TPCP_P5 | ACC | 0.0002812 |
| 1718 | TPCP_P5 | ATG | 0.0003189 |
| 1719 | TPCP_P5 | AAG | 0.0032657 |
| 1720 | TPCP_P5 | AAA | 0.0000000 |
| 1721 | TPCP_P5 | ATC | 0.0003271 |
| 1722 | TPCP_P5 | AAC | 0.0002740 |
| 1723 | TPCP_P5 | ATA | 0.0004882 |
| 1724 | TPCP_P5 | AGG | 0.0002637 |
| 1725 | TPCP_P5 | CCT | 0.0003640 |
| 1726 | TPCP_P5 | CTC | 0.0004196 |
| 1727 | TPCP_P5 | AGC | 0.0003076 |
| 1728 | TPCP_P5 | ACA | 0.0002795 |
| 1729 | TPCP_P5 | AGA | 0.0017845 |
| 1730 | TPCP_P5 | CAT | 0.0005282 |
| 1731 | TPCP_P5 | AAT | 0.0003356 |
| 1732 | TPCP_P5 | ATT | 0.0002892 |
| 1733 | TPCP_P5 | CTG | 0.0003069 |
| 1734 | TPCP_P5 | CTA | 0.0003196 |
| 1735 | TPCP_P5 | ACT | 0.0004804 |
| 1736 | TPCP_P5 | CAC | 0.0002883 |
| 1737 | TPCP_P5 | ACG | 0.0003578 |
| 1738 | TPCP_P5 | CAA | 0.0002983 |
| 1739 | TPCP_P5 | AGT | 0.0058986 |
| 1740 | TPCP_P5 | CAG | 0.0003453 |
| 1741 | TPCP_P5 | CCG | 0.0003118 |
| 1742 | TPCP_P5 | CCC | 0.0003304 |
| 1743 | TPCP_P5 | CTT | 0.0002682 |
| 1744 | TPCP_P5 | TAT | 0.0004239 |
| 1745 | TPCP_P5 | GGT | 0.0002798 |
| 1746 | TPCP_P5 | TGT | 0.0009081 |
| 1747 | TPCP_P5 | CGA | 0.0003179 |
| 1748 | TPCP_P5 | CCA | 0.0055559 |
| 1749 | TPCP_P5 | TCT | 0.0002665 |
| 1750 | TPCP_P5 | GAT | 0.0004348 |
| 1751 | TPCP_P5 | CGG | 0.0002738 |
| 1752 | TPCP_P5 | TTT | 0.0003494 |
| 1753 | TPCP_P5 | TGC | 0.0002790 |
| 1754 | TPCP_P5 | GGG | 0.0003433 |
| 1755 | TPCP_P5 | TAG | 0.0002802 |
| 1756 | TPCP_P5 | GGA | 0.0002472 |
| 1757 | TPCP_P5 | TAA | 0.0002832 |
| 1758 | TPCP_P5 | GGC | 0.0003168 |
| 1759 | TPCP_P5 | TAC | 0.0003953 |
| 1760 | TPCP_P5 | TTC | 0.0005967 |
| 1761 | TPCP_P5 | TCG | 0.0002957 |
| 1762 | TPCP_P5 | TTA | 0.0004710 |
| 1763 | TPCP_P5 | TTG | 0.0003566 |
| 1764 | TPCP_P5 | TCC | 0.0005931 |
| 1765 | TPCP_P5 | GAA | 0.0006445 |
| 1766 | TPCP_P5 | TGG | 0.0004037 |
| 1767 | TPCP_P5 | GCA | 0.0003174 |
| 1768 | TPCP_P5 | GTA | 0.0004844 |
| 1769 | TPCP_P5 | GCC | 0.0009407 |
| 1770 | TPCP_P5 | GTC | 0.0003523 |
| 1771 | TPCP_P5 | GCG | 0.0002932 |
| 1772 | TPCP_P5 | GTG | 0.0006702 |
| 1773 | TPCP_P5 | GAG | 0.0044551 |
| 1774 | TPCP_P5 | GTT | 0.0003038 |
| 1775 | TPCP_P5 | GCT | 0.0003022 |
| 1776 | TPCP_P5 | TGA | 0.0003711 |
| 1777 | TPCP_P5 | GAC | 0.0002893 |
| 1778 | TPCP_P5 | CGT | 0.0003929 |
| 1779 | TPCP_P5 | TCA | 0.0014438 |
| 1780 | TPCP_P5 | CGC | 0.0003478 |
| 1781 | TPCP_P6 | ACC | 0.0002829 |
| 1782 | TPCP_P6 | ATG | 0.0003255 |
| 1783 | TPCP_P6 | AAG | 0.0040915 |
| 1784 | TPCP_P6 | AAA | 0.0000000 |
| 1785 | TPCP_P6 | ATC | 0.0003223 |
| 1786 | TPCP_P6 | AAC | 0.0002730 |
| 1787 | TPCP_P6 | ATA | 0.0004863 |
| 1788 | TPCP_P6 | AGG | 0.0002640 |
| 1789 | TPCP_P6 | CCT | 0.0003582 |
| 1790 | TPCP_P6 | CTC | 0.0004259 |
| 1791 | TPCP_P6 | AGC | 0.0003064 |
| 1792 | TPCP_P6 | ACA | 0.0002812 |
| 1793 | TPCP_P6 | AGA | 0.0011681 |
| 1794 | TPCP_P6 | CAT | 0.0005204 |
| 1795 | TPCP_P6 | AAT | 0.0003345 |
| 1796 | TPCP_P6 | ATT | 0.0002896 |
| 1797 | TPCP_P6 | CTG | 0.0003086 |
| 1798 | TPCP_P6 | CTA | 0.0003206 |
| 1799 | TPCP_P6 | ACT | 0.0004827 |
| 1800 | TPCP_P6 | CAC | 0.0002887 |
| 1801 | TPCP_P6 | ACG | 0.0003952 |
| 1802 | TPCP_P6 | CAA | 0.0002990 |
| 1803 | TPCP_P6 | AGT | 0.0045683 |
| 1804 | TPCP_P6 | CAG | 0.0003437 |
| 1805 | TPCP_P6 | CCG | 0.0003121 |
| 1806 | TPCP_P6 | CCC | 0.0003281 |
| 1807 | TPCP_P6 | CTT | 0.0002667 |
| 1808 | TPCP_P6 | TAT | 0.0003869 |
| 1809 | TPCP_P6 | GGT | 0.0002799 |
| 1810 | TPCP_P6 | TGT | 0.0006137 |
| 1811 | TPCP_P6 | CGA | 0.0003169 |
| 1812 | TPCP_P6 | CCA | 0.0057622 |
| 1813 | TPCP_P6 | TCT | 0.0002668 |
| 1814 | TPCP_P6 | GAT | 0.0004947 |
| 1815 | TPCP_P6 | CGG | 0.0002734 |
| 1816 | TPCP_P6 | TTT | 0.0003439 |
| 1817 | TPCP_P6 | TGC | 0.0002782 |
| 1818 | TPCP_P6 | GGG | 0.0003447 |
| 1819 | TPCP_P6 | TAG | 0.0002603 |
| 1820 | TPCP_P6 | GGA | 0.0002457 |
| 1821 | TPCP_P6 | TAA | 0.0002853 |
| 1822 | TPCP_P6 | GGC | 0.0003151 |
| 1823 | TPCP_P6 | TAC | 0.0003955 |
| 1824 | TPCP_P6 | TTC | 0.0005230 |
| 1825 | TPCP_P6 | TCG | 0.0002961 |
| 1826 | TPCP_P6 | TTA | 0.0005269 |
| 1827 | TPCP_P6 | TTG | 0.0003604 |
| 1828 | TPCP_P6 | TCC | 0.0006097 |
| 1829 | TPCP_P6 | GAA | 0.0006288 |
| 1830 | TPCP_P6 | TGG | 0.0004103 |
| 1831 | TPCP_P6 | GCA | 0.0003175 |
| 1832 | TPCP_P6 | GTA | 0.0004912 |
| 1833 | TPCP_P6 | GCC | 0.0007155 |
| 1834 | TPCP_P6 | GTC | 0.0003527 |
| 1835 | TPCP_P6 | GCG | 0.0002932 |
| 1836 | TPCP_P6 | GTG | 0.0006734 |
| 1837 | TPCP_P6 | GAG | 0.0026877 |
| 1838 | TPCP_P6 | GTT | 0.0003016 |
| 1839 | TPCP_P6 | GCT | 0.0003045 |
| 1840 | TPCP_P6 | TGA | 0.0003492 |
| 1841 | TPCP_P6 | GAC | 0.0002921 |
| 1842 | TPCP_P6 | CGT | 0.0004125 |
| 1843 | TPCP_P6 | TCA | 0.0014560 |
| 1844 | TPCP_P6 | CGC | 0.0003460 |
| 1845 | TPCP_P7 | ACC | 0.0002798 |
| 1846 | TPCP_P7 | ATG | 0.0003183 |
| 1847 | TPCP_P7 | AAG | 0.0032529 |
| 1848 | TPCP_P7 | AAA | 0.0002798 |
| 1849 | TPCP_P7 | ATC | 0.0003269 |
| 1850 | TPCP_P7 | AAC | 0.0002749 |
| 1851 | TPCP_P7 | ATA | 0.0004873 |
| 1852 | TPCP_P7 | AGG | 0.0002630 |
| 1853 | TPCP_P7 | CCT | 0.0003701 |
| 1854 | TPCP_P7 | CTC | 0.0004502 |
| 1855 | TPCP_P7 | AGC | 0.0002998 |
| 1856 | TPCP_P7 | ACA | 0.0002818 |
| 1857 | TPCP_P7 | AGA | 0.0013202 |
| 1858 | TPCP_P7 | CAT | 0.0005265 |
| 1859 | TPCP_P7 | AAT | 0.0003314 |
| 1860 | TPCP_P7 | ATT | 0.0002886 |
| 1861 | TPCP_P7 | CTG | 0.0003079 |
| 1862 | TPCP_P7 | CTA | 0.0003218 |
| 1863 | TPCP_P7 | ACT | 0.0004800 |
| 1864 | TPCP_P7 | CAC | 0.0002888 |
| 1865 | TPCP_P7 | ACG | 0.0003173 |
| 1866 | TPCP_P7 | CAA | 0.0002992 |
| 1867 | TPCP_P7 | AGT | 0.0047781 |
| 1868 | TPCP_P7 | CAG | 0.0003434 |
| 1869 | TPCP_P7 | CCG | 0.0003108 |
| 1870 | TPCP_P7 | CCC | 0.0003285 |
| 1871 | TPCP_P7 | CTT | 0.0002709 |
| 1872 | TPCP_P7 | TAT | 0.0000000 |
| 1873 | TPCP_P7 | GGT | 0.0002813 |
| 1874 | TPCP_P7 | TGT | 0.0007358 |
| 1875 | TPCP_P7 | CGA | 0.0000000 |
| 1876 | TPCP_P7 | CCA | 0.0042991 |
| 1877 | TPCP_P7 | TCT | 0.0002681 |
| 1878 | TPCP_P7 | GAT | 0.0000000 |
| 1879 | TPCP_P7 | CGG | 0.0000000 |
| 1880 | TPCP_P7 | TTT | 0.0000000 |
| 1881 | TPCP_P7 | TGC | 0.0000000 |
| 1882 | TPCP_P7 | GGG | 0.0000000 |
| 1883 | TPCP_P7 | TAG | 0.0000000 |
| 1884 | TPCP_P7 | GGA | 0.0002441 |
| 1885 | TPCP_P7 | TAA | 0.0002833 |
| 1886 | TPCP_P7 | GGC | 0.0000000 |
| 1887 | TPCP_P7 | TAC | 0.0003947 |
| 1888 | TPCP_P7 | TTC | 0.0005443 |
| 1889 | TPCP_P7 | TCG | 0.0002993 |
| 1890 | TPCP_P7 | TTA | 0.0000000 |
| 1891 | TPCP_P7 | TTG | 0.0000000 |
| 1892 | TPCP_P7 | TCC | 0.0000000 |
| 1893 | TPCP_P7 | GAA | 0.0000000 |
| 1894 | TPCP_P7 | TGG | 0.0000000 |
| 1895 | TPCP_P7 | GCA | 0.0003163 |
| 1896 | TPCP_P7 | GTA | 0.0000000 |
| 1897 | TPCP_P7 | GCC | 0.0006897 |
| 1898 | TPCP_P7 | GTC | 0.0000000 |
| 1899 | TPCP_P7 | GCG | 0.0002907 |
| 1900 | TPCP_P7 | GTG | 0.0000000 |
| 1901 | TPCP_P7 | GAG | 0.0029009 |
| 1902 | TPCP_P7 | GTT | 0.0003016 |
| 1903 | TPCP_P7 | GCT | 0.0000000 |
| 1904 | TPCP_P7 | TGA | 0.0000000 |
| 1905 | TPCP_P7 | GAC | 0.0002920 |
| 1906 | TPCP_P7 | CGT | 0.0004202 |
| 1907 | TPCP_P7 | TCA | 0.0011559 |
| 1908 | TPCP_P7 | CGC | 0.0003463 |
| 1909 | TPCP_P8 | ACC | 0.0002802 |
| 1910 | TPCP_P8 | ATG | 0.0000000 |
| 1911 | TPCP_P8 | AAG | 0.0032372 |
| 1912 | TPCP_P8 | AAA | 0.0000000 |
| 1913 | TPCP_P8 | ATC | 0.0003264 |
| 1914 | TPCP_P8 | AAC | 0.0002744 |
| 1915 | TPCP_P8 | ATA | 0.0000000 |
| 1916 | TPCP_P8 | AGG | 0.0002614 |
| 1917 | TPCP_P8 | CCT | 0.0000000 |
| 1918 | TPCP_P8 | CTC | 0.0004026 |
| 1919 | TPCP_P8 | AGC | 0.0000000 |
| 1920 | TPCP_P8 | ACA | 0.0000000 |
| 1921 | TPCP_P8 | AGA | 0.0011248 |
| 1922 | TPCP_P8 | CAT | 0.0000000 |
| 1923 | TPCP_P8 | AAT | 0.0000000 |
| 1924 | TPCP_P8 | ATT | 0.0002930 |
| 1925 | TPCP_P8 | CTG | 0.0000000 |
| 1926 | TPCP_P8 | CTA | 0.0003205 |
| 1927 | TPCP_P8 | ACT | 0.0004806 |
| 1928 | TPCP_P8 | CAC | 0.0000000 |
| 1929 | TPCP_P8 | ACG | 0.0003593 |
| 1930 | TPCP_P8 | CAA | 0.0000000 |
| 1931 | TPCP_P8 | AGT | 0.0052784 |
| 1932 | TPCP_P8 | CAG | 0.0000000 |
| 1933 | TPCP_P8 | CCG | 0.0000000 |
| 1934 | TPCP_P8 | CCC | 0.0000000 |
| 1935 | TPCP_P8 | CTT | 0.0002755 |
| 1936 | TPCP_P8 | TAT | 0.0004347 |
| 1937 | TPCP_P8 | GGT | 0.0000000 |
| 1938 | TPCP_P8 | TGT | 0.0006329 |
| 1939 | TPCP_P8 | CGA | 0.0003596 |
| 1940 | TPCP_P8 | CCA | 0.0048332 |
| 1941 | TPCP_P8 | TCT | 0.0002675 |
| 1942 | TPCP_P8 | GAT | 0.0000000 |
| 1943 | TPCP_P8 | CGG | 0.0000000 |
| 1944 | TPCP_P8 | TTT | 0.0000000 |
| 1945 | TPCP_P8 | TGC | 0.0000000 |
| 1946 | TPCP_P8 | GGG | 0.0000000 |
| 1947 | TPCP_P8 | TAG | 0.0000000 |
| 1948 | TPCP_P8 | GGA | 0.0002454 |
| 1949 | TPCP_P8 | TAA | 0.0002856 |
| 1950 | TPCP_P8 | GGC | 0.0000000 |
| 1951 | TPCP_P8 | TAC | 0.0003964 |
| 1952 | TPCP_P8 | TTC | 0.0005767 |
| 1953 | TPCP_P8 | TCG | 0.0002994 |
| 1954 | TPCP_P8 | TTA | 0.0000000 |
| 1955 | TPCP_P8 | TTG | 0.0000000 |
| 1956 | TPCP_P8 | TCC | 0.0000000 |
| 1957 | TPCP_P8 | GAA | 0.0000000 |
| 1958 | TPCP_P8 | TGG | 0.0000000 |
| 1959 | TPCP_P8 | GCA | 0.0003180 |
| 1960 | TPCP_P8 | GTA | 0.0000000 |
| 1961 | TPCP_P8 | GCC | 0.0006633 |
| 1962 | TPCP_P8 | GTC | 0.0000000 |
| 1963 | TPCP_P8 | GCG | 0.0003036 |
| 1964 | TPCP_P8 | GTG | 0.0000000 |
| 1965 | TPCP_P8 | GAG | 0.0024625 |
| 1966 | TPCP_P8 | GTT | 0.0003044 |
| 1967 | TPCP_P8 | GCT | 0.0000000 |
| 1968 | TPCP_P8 | TGA | 0.0000000 |
| 1969 | TPCP_P8 | GAC | 0.0002900 |
| 1970 | TPCP_P8 | CGT | 0.0004170 |
| 1971 | TPCP_P8 | TCA | 0.0009746 |
| 1972 | TPCP_P8 | CGC | 0.0003444 |
| 1973 | TPCP_P9 | ACC | 0.0002821 |
| 1974 | TPCP_P9 | ATG | 0.0000000 |
| 1975 | TPCP_P9 | AAG | 0.0029332 |
| 1976 | TPCP_P9 | AAA | 0.0000000 |
| 1977 | TPCP_P9 | ATC | 0.0003266 |
| 1978 | TPCP_P9 | AAC | 0.0002756 |
| 1979 | TPCP_P9 | ATA | 0.0000000 |
| 1980 | TPCP_P9 | AGG | 0.0002603 |
| 1981 | TPCP_P9 | CCT | 0.0000000 |
| 1982 | TPCP_P9 | CTC | 0.0004141 |
| 1983 | TPCP_P9 | AGC | 0.0000000 |
| 1984 | TPCP_P9 | ACA | 0.0000000 |
| 1985 | TPCP_P9 | AGA | 0.0011943 |
| 1986 | TPCP_P9 | CAT | 0.0000000 |
| 1987 | TPCP_P9 | AAT | 0.0000000 |
| 1988 | TPCP_P9 | ATT | 0.0002882 |
| 1989 | TPCP_P9 | CTG | 0.0000000 |
| 1990 | TPCP_P9 | CTA | 0.0003178 |
| 1991 | TPCP_P9 | ACT | 0.0004766 |
| 1992 | TPCP_P9 | CAC | 0.0000000 |
| 1993 | TPCP_P9 | ACG | 0.0003322 |
| 1994 | TPCP_P9 | CAA | 0.0000000 |
| 1995 | TPCP_P9 | AGT | 0.0064793 |
| 1996 | TPCP_P9 | CAG | 0.0000000 |
| 1997 | TPCP_P9 | CCG | 0.0000000 |
| 1998 | TPCP_P9 | CCC | 0.0000000 |
| 1999 | TPCP_P9 | CTT | 0.0002684 |
| 2000 | TPCP_P9 | TAT | 0.0003764 |
| 2001 | TPCP_P9 | GGT | 0.0000000 |
| 2002 | TPCP_P9 | TGT | 0.0008119 |
| 2003 | TPCP_P9 | CGA | 0.0003152 |
| 2004 | TPCP_P9 | CCA | 0.0036919 |
| 2005 | TPCP_P9 | TCT | 0.0002668 |
| 2006 | TPCP_P9 | GAT | 0.0004537 |
| 2007 | TPCP_P9 | CGG | 0.0002739 |
| 2008 | TPCP_P9 | TTT | 0.0000000 |
| 2009 | TPCP_P9 | TGC | 0.0002834 |
| 2010 | TPCP_P9 | GGG | 0.0003425 |
| 2011 | TPCP_P9 | TAG | 0.0002757 |
| 2012 | TPCP_P9 | GGA | 0.0002455 |
| 2013 | TPCP_P9 | TAA | 0.0002844 |
| 2014 | TPCP_P9 | GGC | 0.0003163 |
| 2015 | TPCP_P9 | TAC | 0.0003927 |
| 2016 | TPCP_P9 | TTC | 0.0005725 |
| 2017 | TPCP_P9 | TCG | 0.0003076 |
| 2018 | TPCP_P9 | TTA | 0.0004457 |
| 2019 | TPCP_P9 | TTG | 0.0003552 |
| 2020 | TPCP_P9 | TCC | 0.0006186 |
| 2021 | TPCP_P9 | GAA | 0.0006401 |
| 2022 | TPCP_P9 | TGG | 0.0003999 |
| 2023 | TPCP_P9 | GCA | 0.0003186 |
| 2024 | TPCP_P9 | GTA | 0.0004917 |
| 2025 | TPCP_P9 | GCC | 0.0009028 |
| 2026 | TPCP_P9 | GTC | 0.0003510 |
| 2027 | TPCP_P9 | GCG | 0.0002936 |
| 2028 | TPCP_P9 | GTG | 0.0006617 |
| 2029 | TPCP_P9 | GAG | 0.0018982 |
| 2030 | TPCP_P9 | GTT | 0.0002972 |
| 2031 | TPCP_P9 | GCT | 0.0003034 |
| 2032 | TPCP_P9 | TGA | 0.0003673 |
| 2033 | TPCP_P9 | GAC | 0.0002884 |
| 2034 | TPCP_P9 | CGT | 0.0003978 |
| 2035 | TPCP_P9 | TCA | 0.0013693 |
| 2036 | TPCP_P9 | CGC | 0.0003457 |
| 2037 | TPCP_P10 | ACC | 0.0002828 |
| 2038 | TPCP_P10 | ATG | 0.0003198 |
| 2039 | TPCP_P10 | AAG | 0.0042532 |
| 2040 | TPCP_P10 | AAA | 0.0000000 |
| 2041 | TPCP_P10 | ATC | 0.0003256 |
| 2042 | TPCP_P10 | AAC | 0.0002737 |
| 2043 | TPCP_P10 | ATA | 0.0004895 |
| 2044 | TPCP_P10 | AGG | 0.0002606 |
| 2045 | TPCP_P10 | CCT | 0.0003604 |
| 2046 | TPCP_P10 | CTC | 0.0004507 |
| 2047 | TPCP_P10 | AGC | 0.0003036 |
| 2048 | TPCP_P10 | ACA | 0.0002823 |
| 2049 | TPCP_P10 | AGA | 0.0014803 |
| 2050 | TPCP_P10 | CAT | 0.0005266 |
| 2051 | TPCP_P10 | AAT | 0.0003348 |
| 2052 | TPCP_P10 | ATT | 0.0002904 |
| 2053 | TPCP_P10 | CTG | 0.0003079 |
| 2054 | TPCP_P10 | CTA | 0.0003223 |
| 2055 | TPCP_P10 | ACT | 0.0004796 |
| 2056 | TPCP_P10 | CAC | 0.0002891 |
| 2057 | TPCP_P10 | ACG | 0.0003232 |
| 2058 | TPCP_P10 | CAA | 0.0002997 |
| 2059 | TPCP_P10 | AGT | 0.0055932 |
| 2060 | TPCP_P10 | CAG | 0.0003432 |
| 2061 | TPCP_P10 | CCG | 0.0003095 |
| 2062 | TPCP_P10 | CCC | 0.0003294 |
| 2063 | TPCP_P10 | CTT | 0.0002687 |
| 2064 | TPCP_P10 | TAT | 0.0003753 |
| 2065 | TPCP_P10 | GGT | 0.0002795 |
| 2066 | TPCP_P10 | TGT | 0.0006174 |
| 2067 | TPCP_P10 | CGA | 0.0003130 |
| 2068 | TPCP_P10 | CCA | 0.0050596 |
| 2069 | TPCP_P10 | TCT | 0.0002675 |
| 2070 | TPCP_P10 | GAT | 0.0004457 |
| 2071 | TPCP_P10 | CGG | 0.0002740 |
| 2072 | TPCP_P10 | TTT | 0.0003522 |
| 2073 | TPCP_P10 | TGC | 0.0002789 |
| 2074 | TPCP_P10 | GGG | 0.0003460 |
| 2075 | TPCP_P10 | TAG | 0.0002645 |
| 2076 | TPCP_P10 | GGA | 0.0002424 |
| 2077 | TPCP_P10 | TAA | 0.0002838 |
| 2078 | TPCP_P10 | GGC | 0.0003157 |
| 2079 | TPCP_P10 | TAC | 0.0003940 |
| 2080 | TPCP_P10 | TTC | 0.0005426 |
| 2081 | TPCP_P10 | TCG | 0.0002979 |
| 2082 | TPCP_P10 | TTA | 0.0005295 |
| 2083 | TPCP_P10 | TTG | 0.0003641 |
| 2084 | TPCP_P10 | TCC | 0.0006041 |
| 2085 | TPCP_P10 | GAA | 0.0006480 |
| 2086 | TPCP_P10 | TGG | 0.0004074 |
| 2087 | TPCP_P10 | GCA | 0.0003196 |
| 2088 | TPCP_P10 | GTA | 0.0004943 |
| 2089 | TPCP_P10 | GCC | 0.0008632 |
| 2090 | TPCP_P10 | GTC | 0.0003516 |
| 2091 | TPCP_P10 | GCG | 0.0002942 |
| 2092 | TPCP_P10 | GTG | 0.0006637 |
| 2093 | TPCP_P10 | GAG | 0.0033364 |
| 2094 | TPCP_P10 | GTT | 0.0002978 |
| 2095 | TPCP_P10 | GCT | 0.0003045 |
| 2096 | TPCP_P10 | TGA | 0.0003524 |
| 2097 | TPCP_P10 | GAC | 0.0002921 |
| 2098 | TPCP_P10 | CGT | 0.0004023 |
| 2099 | TPCP_P10 | TCA | 0.0014372 |
| 2100 | TPCP_P10 | CGC | 0.0003474 |
| 2101 | TPCP_P11 | ACC | 0.0002784 |
| 2102 | TPCP_P11 | ATG | 0.0003332 |
| 2103 | TPCP_P11 | AAG | 0.0032664 |
| 2104 | TPCP_P11 | AAA | 0.0002670 |
| 2105 | TPCP_P11 | ATC | 0.0003259 |
| 2106 | TPCP_P11 | AAC | 0.0002726 |
| 2107 | TPCP_P11 | ATA | 0.0004914 |
| 2108 | TPCP_P11 | AGG | 0.0002641 |
| 2109 | TPCP_P11 | CCT | 0.0003630 |
| 2110 | TPCP_P11 | CTC | 0.0000000 |
| 2111 | TPCP_P11 | AGC | 0.0003023 |
| 2112 | TPCP_P11 | ACA | 0.0002806 |
| 2113 | TPCP_P11 | AGA | 0.0012729 |
| 2114 | TPCP_P11 | CAT | 0.0005295 |
| 2115 | TPCP_P11 | AAT | 0.0003341 |
| 2116 | TPCP_P11 | ATT | 0.0002919 |
| 2117 | TPCP_P11 | CTG | 0.0003097 |
| 2118 | TPCP_P11 | CTA | 0.0003211 |
| 2119 | TPCP_P11 | ACT | 0.0004758 |
| 2120 | TPCP_P11 | CAC | 0.0002894 |
| 2121 | TPCP_P11 | ACG | 0.0000000 |
| 2122 | TPCP_P11 | CAA | 0.0002997 |
| 2123 | TPCP_P11 | AGT | 0.0051233 |
| 2124 | TPCP_P11 | CAG | 0.0003431 |
| 2125 | TPCP_P11 | CCG | 0.0003127 |
| 2126 | TPCP_P11 | CCC | 0.0003292 |
| 2127 | TPCP_P11 | CTT | 0.0002711 |
| 2128 | TPCP_P11 | TAT | 0.0004729 |
| 2129 | TPCP_P11 | GGT | 0.0002799 |
| 2130 | TPCP_P11 | TGT | 0.0007234 |
| 2131 | TPCP_P11 | CGA | 0.0003100 |
| 2132 | TPCP_P11 | CCA | 0.0058612 |
| 2133 | TPCP_P11 | TCT | 0.0003815 |
| 2134 | TPCP_P11 | GAT | 0.0002954 |
| 2135 | TPCP_P11 | CGG | 0.0003046 |
| 2136 | TPCP_P11 | TTT | 0.0002679 |
| 2137 | TPCP_P11 | TGC | 0.0003507 |
| 2138 | TPCP_P11 | GGG | 0.0004077 |
| 2139 | TPCP_P11 | TAG | 0.0000000 |
| 2140 | TPCP_P11 | GGA | 0.0003149 |
| 2141 | TPCP_P11 | TAA | 0.0008445 |
| 2142 | TPCP_P11 | GGC | 0.0003964 |
| 2143 | TPCP_P11 | TAC | 0.0002724 |
| 2144 | TPCP_P11 | TTC | 0.0006260 |
| 2145 | TPCP_P11 | TCG | 0.0002701 |
| 2146 | TPCP_P11 | TTA | 0.0011473 |
| 2147 | TPCP_P11 | TTG | 0.0002553 |
| 2148 | TPCP_P11 | TCC | 0.0000000 |
| 2149 | TPCP_P11 | GAA | 0.0003682 |
| 2150 | TPCP_P11 | TGG | 0.0002955 |
| 2151 | TPCP_P11 | GCA | 0.0003031 |
| 2152 | TPCP_P11 | GTA | 0.0002664 |
| 2153 | TPCP_P11 | GCC | 0.0003593 |
| 2154 | TPCP_P11 | GTC | 0.0004118 |
| 2155 | TPCP_P11 | GCG | 0.0067044 |
| 2156 | TPCP_P11 | GTG | 0.0003218 |
| 2157 | TPCP_P11 | GAG | 0.0010731 |
| 2158 | TPCP_P11 | GTT | 0.0004399 |
| 2159 | TPCP_P11 | GCT | 0.0002716 |
| 2160 | TPCP_P11 | TGA | 0.0006202 |
| 2161 | TPCP_P11 | GAC | 0.0002718 |
| 2162 | TPCP_P11 | CGT | 0.0009301 |
| 2163 | TPCP_P11 | TCA | 0.0002551 |
| 2164 | TPCP_P11 | CGC | 0.0000000 |
| 2165 | TPCP_P12 | ACC | 0.0003757 |
| 2166 | TPCP_P12 | ATG | 0.0002973 |
| 2167 | TPCP_P12 | AAG | 0.0003057 |
| 2168 | TPCP_P12 | AAA | 0.0002692 |
| 2169 | TPCP_P12 | ATC | 0.0003617 |
| 2170 | TPCP_P12 | AAC | 0.0004409 |
| 2171 | TPCP_P12 | ATA | 0.0060669 |
| 2172 | TPCP_P12 | AGG | 0.0003175 |
| 2173 | TPCP_P12 | CCT | 0.0013860 |
| 2174 | TPCP_P12 | CTC | 0.0004068 |
| 2175 | TPCP_P12 | AGC | 0.0002737 |
| 2176 | TPCP_P12 | ACA | 0.0006282 |
| 2177 | TPCP_P12 | AGA | 0.0002718 |
| 2178 | TPCP_P12 | CAT | 0.0009061 |
| 2179 | TPCP_P12 | AAT | 0.0002569 |
| 2180 | TPCP_P12 | ATT | 0.0000000 |
| 2181 | TPCP_P12 | CTG | 0.0003712 |
| 2182 | TPCP_P12 | CTA | 0.0002963 |
| 2183 | TPCP_P12 | ACT | 0.0003031 |
| 2184 | TPCP_P12 | CAC | 0.0002688 |
| 2185 | TPCP_P12 | ACG | 0.0003517 |
| 2186 | TPCP_P12 | CAA | 0.0004207 |
| 2187 | TPCP_P12 | AGT | 0.0000000 |
| 2188 | TPCP_P12 | CAG | 0.0003207 |
| 2189 | TPCP_P12 | CCG | 0.0012453 |
| 2190 | TPCP_P12 | CCC | 0.0003877 |
| 2191 | TPCP_P12 | CTT | 0.0002714 |
| 2192 | TPCP_P12 | TAT | 0.0006389 |
| 2193 | TPCP_P12 | GGT | 0.0002731 |
| 2194 | TPCP_P12 | TGT | 0.0009103 |
| 2195 | TPCP_P12 | CGA | 0.0002548 |
| 2196 | TPCP_P12 | CCA | 0.0007480 |
| 2197 | TPCP_P12 | TCT | 0.0003843 |
| 2198 | TPCP_P12 | GAT | 0.0002986 |
| 2199 | TPCP_P12 | CGG | 0.0003056 |
| 2200 | TPCP_P12 | TTT | 0.0002684 |
| 2201 | TPCP_P12 | TGC | 0.0003533 |
| 2202 | TPCP_P12 | GGG | 0.0004151 |
| 2203 | TPCP_P12 | TAG | 0.0000000 |
| 2204 | TPCP_P12 | GGA | 0.0003236 |
| 2205 | TPCP_P12 | TAA | 0.0013675 |
| 2206 | TPCP_P12 | GGC | 0.0004241 |
| 2207 | TPCP_P12 | TAC | 0.0002743 |
| 2208 | TPCP_P12 | TTC | 0.0006456 |
| 2209 | TPCP_P12 | TCG | 0.0002718 |
| 2210 | TPCP_P12 | TTA | 0.0009019 |
| 2211 | TPCP_P12 | TTG | 0.0002557 |
| 2212 | TPCP_P12 | TCC | 0.0000000 |
| 2213 | TPCP_P12 | GAA | 0.0003739 |
| 2214 | TPCP_P12 | TGG | 0.0002996 |
| 2215 | TPCP_P12 | GCA | 0.0003031 |
| 2216 | TPCP_P12 | GTA | 0.0002708 |
| 2217 | TPCP_P12 | GCC | 0.0003451 |
| 2218 | TPCP_P12 | GTC | 0.0004357 |
| 2219 | TPCP_P12 | GCG | 0.0061253 |
| 2220 | TPCP_P12 | GTG | 0.0003215 |
| 2221 | TPCP_P12 | GAG | 0.0008957 |
| 2222 | TPCP_P12 | GTT | 0.0004056 |
| 2223 | TPCP_P12 | GCT | 0.0002741 |
| 2224 | TPCP_P12 | TGA | 0.0007846 |
| 2225 | TPCP_P12 | GAC | 0.0002706 |
| 2226 | TPCP_P12 | CGT | 0.0009201 |
| 2227 | TPCP_P12 | TCA | 0.0002544 |
| 2228 | TPCP_P12 | CGC | 0.0000000 |
