# Supplementary material for: DHSpred: support-vector-machine-based human DNase I hypersensitive sites prediction using the optimal features selected by random forest
Source: Oncotarget. 2017 Dec 8;9(2):1944–56. doi: 10.18632/oncotarget.23099 (PMC5788611; doi:10.18632/oncotarget.23099)
Supplement: Supplementary file 3 [file oncotarget-09-1944-s003.docx]

**Supplementary Table 3: The values of 12 DNA trinucleotide physicochemical properties.**

|  | **P1** | **P2** | **P3** | **P4** | **P5** | **P6** | **P7** | **P8** | **P9** | **P10** | **P11** | **P12** |
| --- | --- | --- | --- | --- | --- | --- | --- | --- | --- | --- | --- | --- |
| GGG | 5.70 | 5.85 | 3 | 13 | 5.83 | 5.83 | 3.31 | 3.87 | 622.40 | 103.39 | 6.00 | 3.54 |
| GGA | 6.20 | 5.00 | 2 | $-$5 | 4.99 | 4.99 | 3.82 | 3.58 | 622.40 | 103.39 | 3.80 | 4.80 |
| GGC | 8.20 | 9.10 | 3 | 45 | 9.08 | 9.08 | 1.39 | 2.45 | 622.40 | 103.39 | 10.00 | 1.31 |
| GGT | 5.20 | 5.30 | 2 | 8 | 5.32 | 5.32 | 3.62 | 4.16 | 622.40 | 103.39 | 5.40 | 3.88 |
| GAG | 6.60 | 6.00 | 2 | 8 | 5.98 | 5.98 | 3.22 | 3.35 | 621.40 | 103.22 | 5.40 | 3.88 |
| GAA | 5.10 | 4.05 | 1 | $-$12 | 4.06 | 4.06 | 4.39 | 4.21 | 621.40 | 103.22 | 3.00 | 5.26 |
| GAC | 5.60 | 5.50 | 2 | 8 | 5.52 | 5.52 | 3.50 | 3.93 | 621.40 | 103.22 | 5.40 | 3.88 |
| GAT | 3.60 | 4.45 | 1 | 7 | 4.44 | 4.44 | 4.15 | 5.09 | 621.40 | 103.22 | 5.30 | 3.94 |
| GCG | 4.30 | 5.90 | 3 | 25 | 5.89 | 5.89 | 3.28 | 4.68 | 622.40 | 103.39 | 7.50 | 2.69 |
| GCA | 7.50 | 6.75 | 2 | 13 | 6.76 | 6.76 | 2.75 | 2.84 | 622.40 | 103.39 | 6.00 | 3.54 |
| GCC | 8.20 | 9.10 | 3 | 45 | 9.08 | 9.08 | 1.39 | 2.45 | 622.40 | 103.39 | 10.00 | 1.31 |
| GCT | 6.30 | 6.90 | 2 | 25 | 6.88 | 6.88 | 2.68 | 3.52 | 622.40 | 103.39 | 7.50 | 2.69 |
| GTG | 6.80 | 6.65 | 2 | 17 | 6.63 | 6.63 | 2.83 | 3.24 | 621.40 | 103.22 | 6.50 | 3.25 |
| GTA | 6.40 | 5.05 | 1 | $-$6 | 5.07 | 5.07 | 3.77 | 3.47 | 621.40 | 103.22 | 3.70 | 4.86 |
| GTC | 5.60 | 5.50 | 2 | 8 | 5.52 | 5.52 | 3.50 | 3.93 | 621.40 | 103.22 | 5.40 | 3.88 |
| GTT | 1.60 | 2.65 | 1 | $-$6 | 2.64 | 2.64 | 5.26 | 6.27 | 621.40 | 103.22 | 3.70 | 4.86 |
| AGG | 4.70 | 5.05 | 2 | 8 | 5.05 | 5.05 | 3.78 | 4.45 | 622.40 | 103.39 | 5.40 | 3.88 |
| AGA | 6.50 | 4.90 | 1 | $-$9 | 4.89 | 4.89 | 3.88 | 3.41 | 622.40 | 103.39 | 3.30 | 5.09 |
| AGC | 6.30 | 6.90 | 2 | 25 | 6.88 | 6.88 | 2.68 | 3.52 | 622.40 | 103.39 | 7.50 | 2.69 |
| AGT | 2.00 | 3.90 | 1 | 11 | 3.92 | 3.92 | 4.47 | 6.03 | 622.40 | 103.39 | 5.80 | 3.65 |
| AAG | 4.20 | 4.70 | 1 | 6 | 4.70 | 4.70 | 4.00 | 4.74 | 621.40 | 103.22 | 5.20 | 3.99 |
| AAA | 0.10 | 0.05 | 0 | $-$36 | 0.06 | 0.06 | 6.88 | 7.18 | 621.40 | 103.22 | 0.00 | 7.05 |
| AAC | 1.60 | 2.65 | 1 | $-$6 | 2.64 | 2.64 | 5.26 | 6.27 | 621.40 | 103.22 | 3.70 | 4.86 |
| AAT | 0.00 | 0.35 | 0 | $-$30 | 0.35 | 0.35 | 6.70 | 7.24 | 621.40 | 103.22 | 0.70 | 6.62 |
| ACG | 5.20 | 5.30 | 2 | 8 | 5.31 | 5.31 | 3.63 | 4.16 | 622.40 | 103.39 | 5.40 | 3.88 |
| ACA | 5.80 | 5.50 | 1 | 6 | 5.49 | 5.49 | 3.52 | 3.81 | 622.40 | 103.39 | 5.20 | 3.99 |
| ACC | 5.20 | 5.30 | 2 | 8 | 5.32 | 5.32 | 3.62 | 4.16 | 622.40 | 103.39 | 5.40 | 3.88 |
| ACT | 2.00 | 3.90 | 1 | 11 | 3.92 | 3.92 | 4.47 | 6.03 | 622.40 | 103.39 | 5.80 | 3.65 |
| ATG | 8.70 | 7.70 | 1 | 18 | 7.72 | 7.72 | 2.19 | 2.17 | 621.40 | 103.22 | 6.70 | 3.14 |
| ATA | 9.70 | 6.25 | 0 | $-$13 | 6.27 | 6.27 | 3.05 | 1.61 | 621.40 | 103.22 | 2.80 | 5.38 |
| ATC | 3.60 | 4.45 | 1 | 7 | 4.44 | 4.44 | 4.15 | 5.09 | 621.40 | 103.22 | 5.30 | 3.94 |
| ATT | 0.00 | 0.35 | 0 | $-$30 | 0.35 | 0.35 | 6.70 | 7.24 | 621.40 | 103.22 | 0.70 | 6.62 |
| CGG | 3.00 | 3.85 | 3 | 2 | 3.87 | 3.87 | 4.50 | 5.44 | 622.40 | 103.39 | 4.70 | 4.28 |
| CGA | 5.80 | 7.05 | 2 | 31 | 7.07 | 7.07 | 2.57 | 3.81 | 622.40 | 103.39 | 8.30 | 2.25 |
| CGC | 4.30 | 5.90 | 3 | 25 | 5.89 | 5.89 | 3.28 | 4.68 | 622.40 | 103.39 | 7.50 | 2.69 |
| CGT | 5.20 | 5.30 | 2 | 8 | 5.31 | 5.31 | 3.63 | 4.16 | 622.40 | 103.39 | 5.40 | 3.88 |
| CAG | 9.60 | 6.90 | 2 | $-$2 | 6.90 | 6.90 | 2.67 | 1.67 | 621.40 | 103.22 | 4.20 | 4.57 |
| CAA | 6.20 | 4.75 | 1 | $-$9 | 4.76 | 4.76 | 3.96 | 3.58 | 621.40 | 103.22 | 3.30 | 5.09 |
| CAC | 6.80 | 6.65 | 2 | 17 | 6.63 | 6.63 | 2.83 | 3.24 | 621.40 | 103.22 | 6.50 | 3.25 |
| CAT | 8.70 | 7.70 | 1 | 18 | 7.72 | 7.72 | 2.19 | 2.17 | 621.40 | 103.22 | 6.70 | 3.14 |
| CCG | 3.00 | 3.85 | 3 | 2 | 3.87 | 3.87 | 4.50 | 5.44 | 622.40 | 103.39 | 4.70 | 4.28 |
| CCA | 0.70 | 3.05 | 2 | 8 | 3.06 | 3.06 | 5.00 | 6.81 | 622.40 | 103.39 | 5.40 | 3.88 |
| CCC | 5.70 | 5.85 | 3 | 13 | 5.83 | 5.83 | 3.31 | 3.87 | 622.40 | 103.39 | 6.00 | 3.54 |
| CCT | 4.70 | 5.05 | 2 | 8 | 5.05 | 5.05 | 3.78 | 4.45 | 622.40 | 103.39 | 5.40 | 3.88 |
| CTG | 9.60 | 6.90 | 2 | $-$2 | 6.90 | 6.90 | 2.67 | 1.67 | 621.40 | 103.22 | 4.20 | 4.57 |
| CTA | 7.80 | 5.00 | 1 | $-$18 | 5.00 | 5.00 | 3.81 | 2.67 | 621.40 | 103.22 | 2.20 | 5.73 |
| CTC | 6.60 | 6.00 | 2 | 8 | 5.98 | 5.98 | 3.22 | 3.35 | 621.40 | 103.22 | 5.40 | 3.88 |
| CTT | 4.20 | 4.70 | 1 | 6 | 4.70 | 4.70 | 4.00 | 4.74 | 621.40 | 103.22 | 5.20 | 3.99 |
| TGG | 0.70 | 3.05 | 2 | 8 | 3.06 | 3.06 | 5.00 | 6.81 | 622.40 | 103.39 | 5.40 | 3.88 |
| TGA | 10.00 | 7.70 | 1 | 8 | 7.70 | 7.70 | 10.00 | 1.45 | 622.40 | 103.39 | 5.40 | 3.88 |
| TGC | 7.50 | 6.75 | 2 | 13 | 6.76 | 6.76 | 2.75 | 2.84 | 622.40 | 103.39 | 6.00 | 3.54 |
| TGT | 5.80 | 5.50 | 1 | 6 | 5.49 | 5.49 | 3.52 | 3.81 | 622.40 | 103.39 | 5.20 | 3.99 |
| TAG | 7.80 | 5.00 | 1 | $-$18 | 5.00 | 5.00 | 3.81 | 2.67 | 621.40 | 103.22 | 2.20 | 5.73 |
| TAA | 7.30 | 4.65 | 0 | $-$20 | 4.67 | 4.67 | 4.01 | 2.96 | 621.40 | 103.22 | 2.00 | 5.85 |
| TAC | 6.40 | 5.05 | 1 | $-$6 | 5.07 | 5.07 | 3.77 | 3.47 | 621.40 | 103.22 | 3.70 | 4.86 |
| TAT | 9.70 | 6.25 | 0 | $-$13 | 6.27 | 6.27 | 3.05 | 1.61 | 621.40 | 103.22 | 2.80 | 5.38 |
| TCG | 5.80 | 7.05 | 2 | 31 | 7.07 | 7.07 | 2.57 | 3.81 | 622.40 | 103.39 | 8.30 | 2.25 |
| TCA | 10.00 | 7.70 | 1 | 8 | 7.70 | 7.70 | 2.20 | 1.45 | 622.40 | 103.39 | 5.40 | 3.88 |
| TCC | 6.20 | 5.00 | 2 | $-$5 | 4.99 | 4.99 | 3.82 | 3.58 | 622.40 | 103.39 | 3.80 | 4.80 |
| TCT | 6.50 | 4.90 | 1 | $-$9 | 4.89 | 4.89 | 3.88 | 3.41 | 622.40 | 103.39 | 3.30 | 5.09 |
| TTG | 6.20 | 4.75 | 1 | $-$9 | 4.76 | 4.76 | 3.96 | 3.58 | 621.40 | 103.22 | 3.30 | 5.09 |
| TTA | 7.30 | 4.65 | 0 | $-$20 | 4.67 | 4.67 | 4.01 | 2.96 | 621.40 | 103.22 | 2.00 | 5.85 |
| TTC | 5.10 | 4.05 | 1 | $-$12 | 4.06 | 4.06 | 4.39 | 4.21 | 621.40 | 103.22 | 3.00 | 5.26 |
| TTT | 0.10 | 0.05 | 0 | $-$36 | 0.06 | 0.06 | 0.10 | 7.18 | 621.40 | 103.22 | 0.00 | 7.05 |

The first column represents the trinucleotides. P1: Bendability (DNase); P2: Bendability (consensus); P3: Trinucleotide CG content; P4: Nucleosome positioning; P5: Consensus (roll), P6: Consensus (rigid), P7: DNase I; P8: DNase I (rigid); P9: Molecular weight (Daltons); P10: Molecular weight (kg); P11: Nucleosome; P12: Nucleosome (rigid).
